# Supplementary material for: Double-negative B cells and DNASE1L3 colocalise with microbiota in gut-associated lymphoid tissue
Source: Nat Commun. 2024 May 14;15:4051. doi: 10.1038/s41467-024-48267-4 (PMC11094119; doi:10.1038/s41467-024-48267-4)
Supplement: Supplementary file 1 — Supplementary Information [file 41467_2024_48267_MOESM1_ESM.pdf]

Double-negative B cells and DNASE1L3 colocalise with microbiota in gut associated lymphoid tissue

Montorsi, Pitcher et al

Supplementary Information

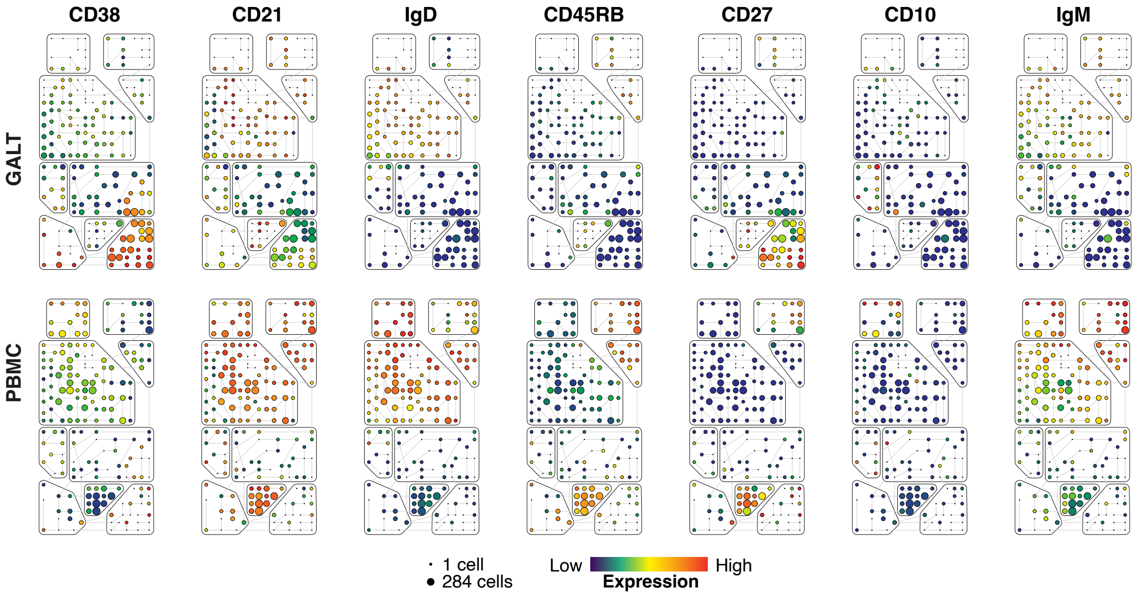

Supplementary Figure 1: Computational analysis of CyTOF data

Representative SPADE plots of matched gut associated lymphoid tissue (GALT) (top row) and peripheral blood mononuclear cells (PBMC) (bottom row) from the same donor (marked green in **Fig. 1f**). Node size indicates number of cells and colour indicates median expression level for each marker.

Figure S2

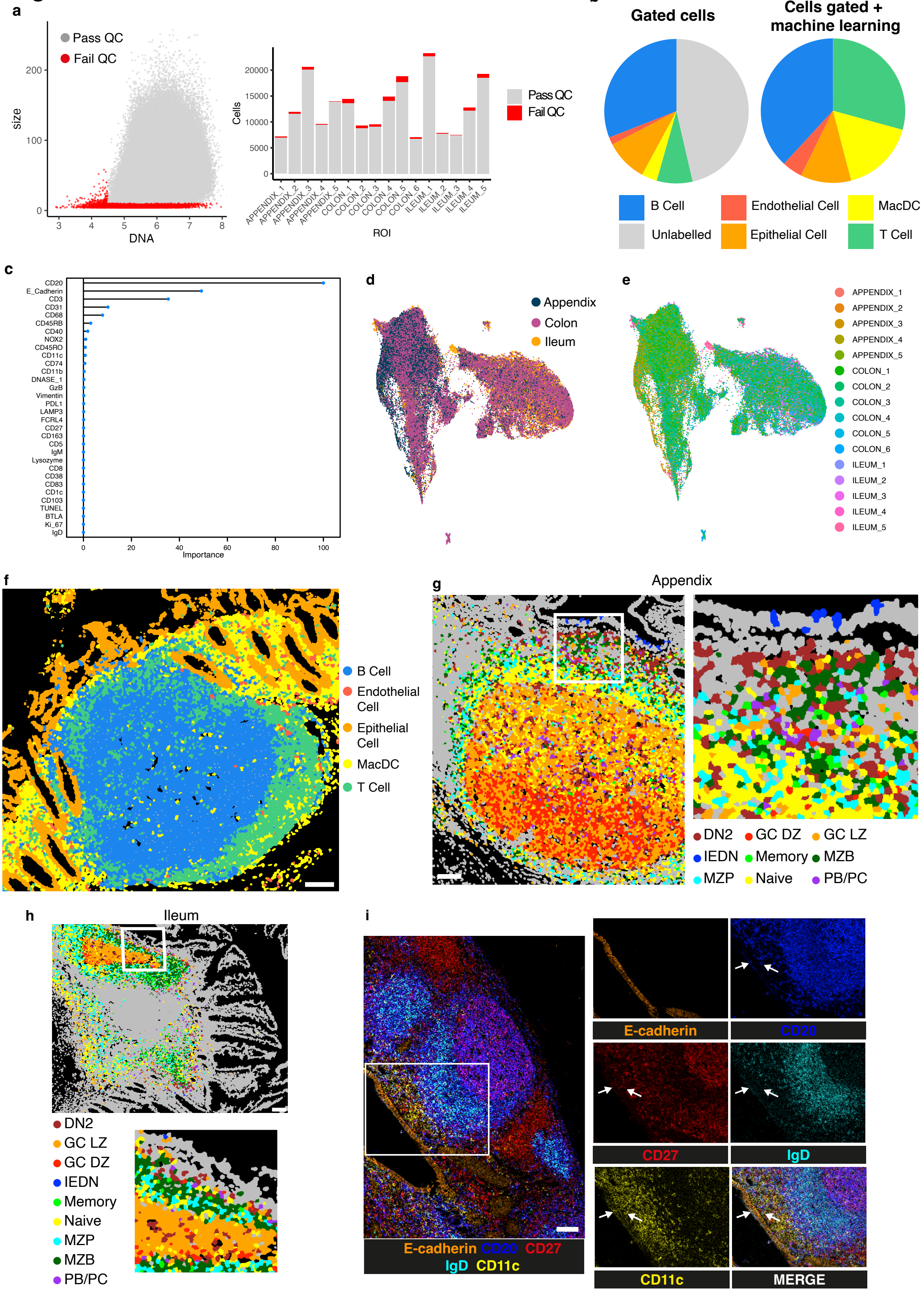
a
Pass QC
Fail QC
size
DNA
Cells
ROI
APPENDIX_1
APPENDIX_2
APPENDIX_3
APPENDIX_4
APPENDIX_5
COLON_1
COLON_2
COLON_3
COLON_4
COLON_5
COLON_6
ILEUM_1
ILEUM_2
ILEUM_3
ILEUM_4
ILEUM_5
b
Gated cells
Cells gated + machine learning
B Cell
Endothelial Cell
MacDC
Unlabelled
Epithelial Cell
T Cell
c
CD20
E_Cadherin
CD3
CD31
CD68
CD45RB
CD40
NOX2
CD45RO
CD11c
CD74
CD11b
DNASE_1
GzB
Vimentin
PDL1
LAMP3
FCRL4
CD27
CD163
CD5
IgM
Lysozyme
CD8
CD38
CD83
CD1c
CD103
TUNEL
BTLA
Ki_67
IgD
Importance
d
Appendix
Colon
Ileum
e
APPENDIX_1
APPENDIX_2
APPENDIX_3
APPENDIX_4
APPENDIX_5
COLON_1
COLON_2
COLON_3
COLON_4
COLON_5
COLON_6
ILEUM_1
ILEUM_2
ILEUM_3
ILEUM_4
ILEUM_5
f
B Cell
Endothelial Cell
Epithelial Cell
MacDC
T Cell
g
Appendix
DN2
GC DZ
GC LZ
IEDN
Memory
MZB
MZP
Naive
PB/PC
h
Ileum
DN2
GC LZ
GC DZ
IEDN
Memory
Naive
MZP
MZB
PB/PC
i
E-cadherin CD20 CD27
IgD CD11c
E-cadherin
CD20
CD27
IgD
CD11c
MERGE

Supplementary Figure 2 Computational analysis of imaging mass cytometry data

a Quality control (QC) of segmented cells on images, removing cells with small size or lack of DNA staining (grey = cells passing quality control, red = cells failing quality control), shown collectively (left) and split by ROI (right). **b** Pie charts showing the proportions of cell types in the gated cells (left), and in the final classifications from gating and machine learning (right). **c** Relative importance of each marker in the IMC panel to the machine learning algorithm. **d** UMAP overlaid with tissue site for each cell. **e** UMAP overlaid with ROI ID for each cell. **f** Overlay of cell types on colon sample shown in **Fig. 2a**. **g** Overlay of B cell subtypes on human appendix sample. **h** Overlay of B cell subtypes on human ileum sample. (DN = double negative, IEDN = intraepithelial DN, PB/PC = plasmablast/plasma cells, MZB = marginal zone B cells, MZP = marginal zone precursor, GC LZ = germinal centre light zone, GC DZ = germinal centre dark zone) **i** Illustrative example of IMC derived image of appendix sample used to validate presence of CD11c+ DN B cells in and near the follicle associated epithelium and sub-epithelial dome. Scalebars = 100μm. Source data are provided as a Source Data file.

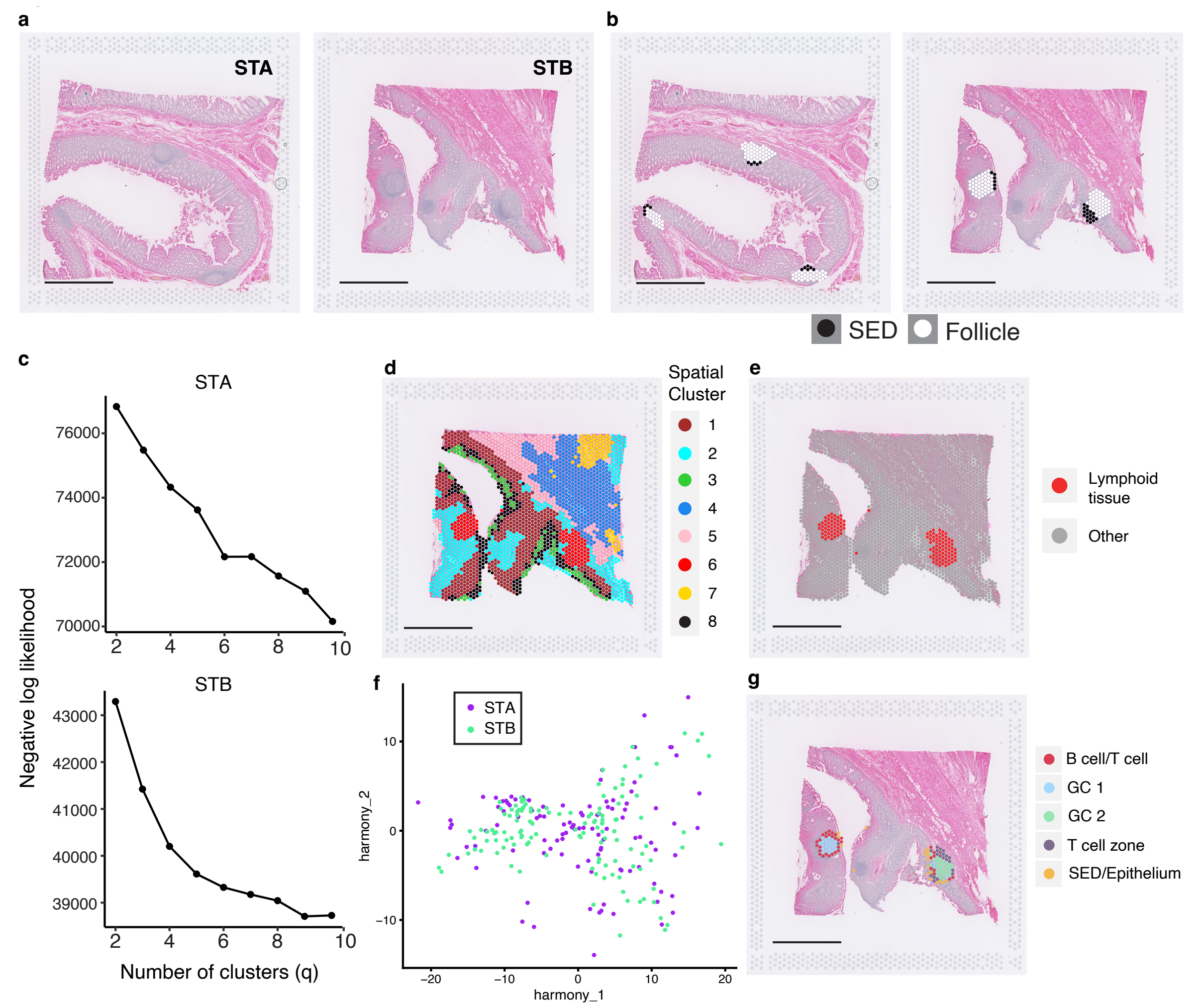

h **Spatial correlation R values**

	C1QA	C1QB	C1QC	C1R	C1S	C3	DNASE1L3	ACP5	AGER	CYBB	DNASE1	FAS
C1QB	0.43											
C1QC	0.32	0.30										
C1R	0.19	0.23	0.24									
C1S	0.10	0.24	0.10	0.21								
C3	0.11	0.22	0.25	0.40	0.18							
DNASE1L3	0.28	0.32	0.28	0.30	0.13	0.32						
ACP5	0.03	0.04	0.03	0.16	0.03	0.15	0.09					
AGER	−0.07	0.04	−0.01	0.05	0.07	0.07	0.04	0.04				
CYBB	−0.02	0.04	−0.10	0.04	0.16	0.05	0.07	−0.06	−0.01			
DNASE1	−0.08	−0.02	0.01	−0.06	0.02	−0.04	−0.09	0.07	−0.02	−0.09		
FAS	0.00	0.13	0.00	0.09	0.01	−0.02	0.12	0.05	0.07	0.11	−0.15	
TMEM173	0.07	0.13	0.09	0.28	0.05	0.22	0.12	0.10	0.05	−0.06	−0.08	0.13

i **Spatial correlation P values**

	C1QA	C1QB	C1QC	C1R	C1S	C3	DNASE1L3	ACP5	AGER	CYBB	DNASE1	FAS
C1QB	0.0001											
C1QC	0.0001	0.0003										
C1R	0.1869	0.0192	0.0111									
C1S	1.0000	0.0109	1.0000	0.0740								
C3	1.0000	0.0445	0.0066	0.0001	0.3460							
DNASE1L3	0.0007	0.0001	0.0009	0.0002	1.0000	0.0001						
ACP5	1.0000	1.0000	1.0000	0.9900	1.0000	1.0000	1.0000					
AGER	1.0000	1.0000	1.0000	1.0000	1.0000	1.0000	1.0000	1.0000				
CYBB	1.0000	1.0000	1.0000	1.0000	0.9721	1.0000	1.0000	1.0000	1.0000			
DNASE1	1.0000	1.0000	1.0000	1.0000	1.0000	1.0000	1.0000	1.0000	1.0000	1.0000		
FAS	1.0000	1.0000	1.0000	1.0000	1.0000	1.0000	1.0000	1.0000	1.0000	1.0000	1.0000	
TMEM173	1.0000	1.0000	1.0000	0.0009	1.0000	0.0528	1.0000	1.0000	1.0000	1.0000	1.0000	1.0000

Supplementary Figure 3: Computational analysis of spatial transcriptomic data

a H&E staining of spatial transcriptomic samples STA and STB. **b** Manual classification of spots as being either in the sub-epithelial dome ('SED', black) or rest of follicle ('Follicle', white). **c**. Negative log-likelihood plot for varying spatial cluster numbers (q) used to determine ideal spatial cluster number per image. **d** Spatial clusters for sample STB. **e** Lymphoid tissue spot assignment for sample STB. **f** Harmonised principal component analysis showing overlap of spots from STA (purple) and STB (green). **g** Sub-cluster regions

for sample STB (GC = germinal centre). **h** Correlation R values for gene correlations as shown in **Fig. 3i. i** Correlation P values for gene correlations as shown in **Fig. 3i**. Scalebars = 2mm. Source data are provided as a Source Data file.

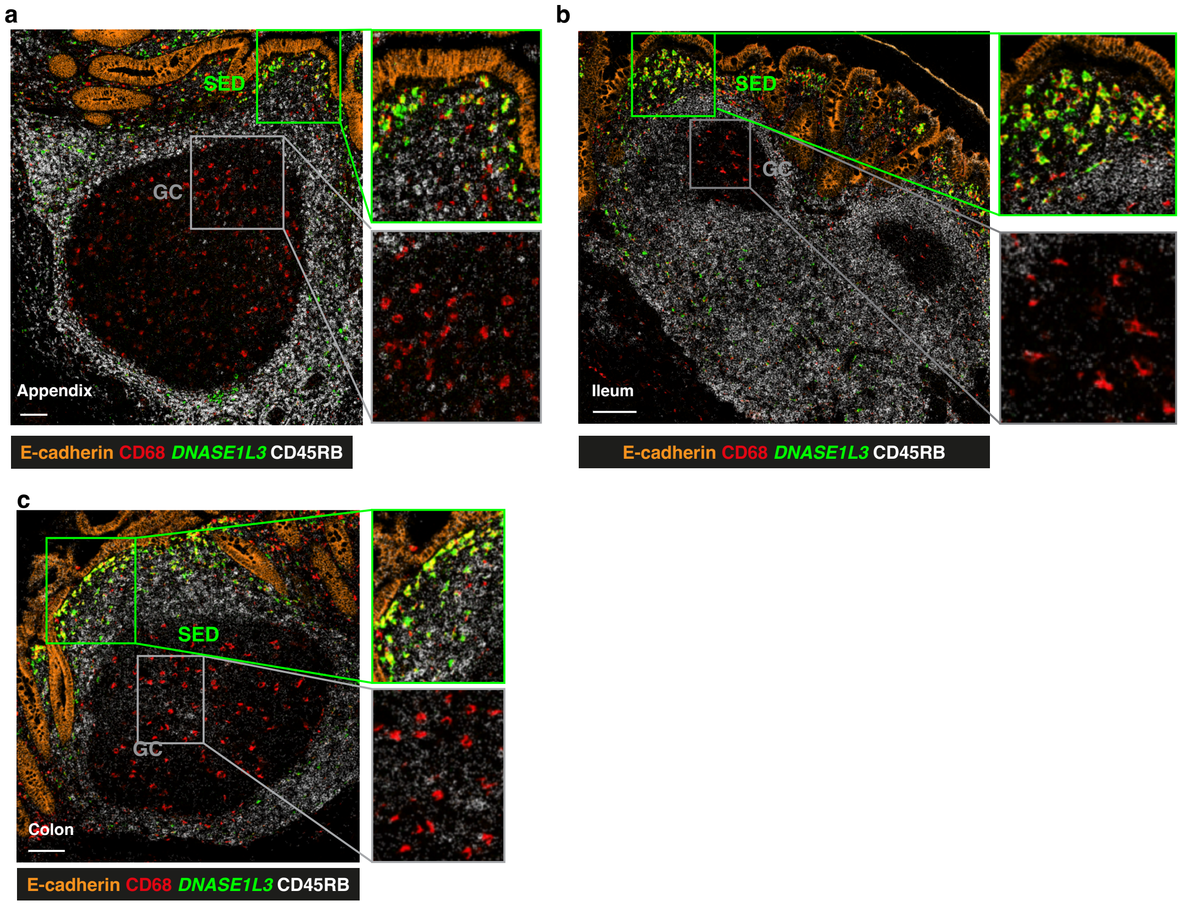

Supplementary Figure 4: DNASE1L3 distribution in Appendix, Ileum and Colon.

Images from IMC coupled with RNAscope analysis showing the distribution of CD68 (red) and CD45RB (white) localization alongside *DNASE1L3* (green) gene expression in **a** Appendix, **b** Ileum, **c** colon. Subepithelial dome (SED) and germinal centre (GC) are highlighted with green and grey boxes respectively. Scalebars = 100μm. Findings are representative of 5 donors (ileum 3 donors, 4 ROI; appendix 3 donors, 5 ROI; colon 2 donors, 7 ROI) in total.

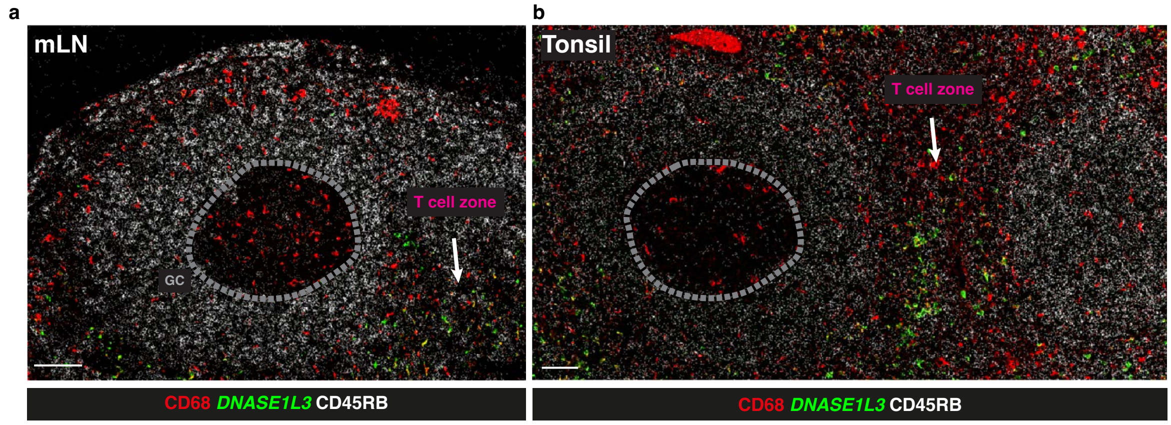

Supplementary Figure 5: DNASE1L3 distribution in mLN and Tonsil

a, b Images from IMC coupled with RNAscope analysis showing the distribution of CD68 (red) and CD45RB (white) localization alongside *DNASE1L3* (green) gene expression in **a** mesenteric lymph node (mLN) and **b** Tonsil. Germinal centre (GC) (grey dashed lines) and T cell zone (magenta) are highlighted. Scalebars = 100μm. Findings are representative of 2 mesenteric lymph node donors and 2 tonsils donors.

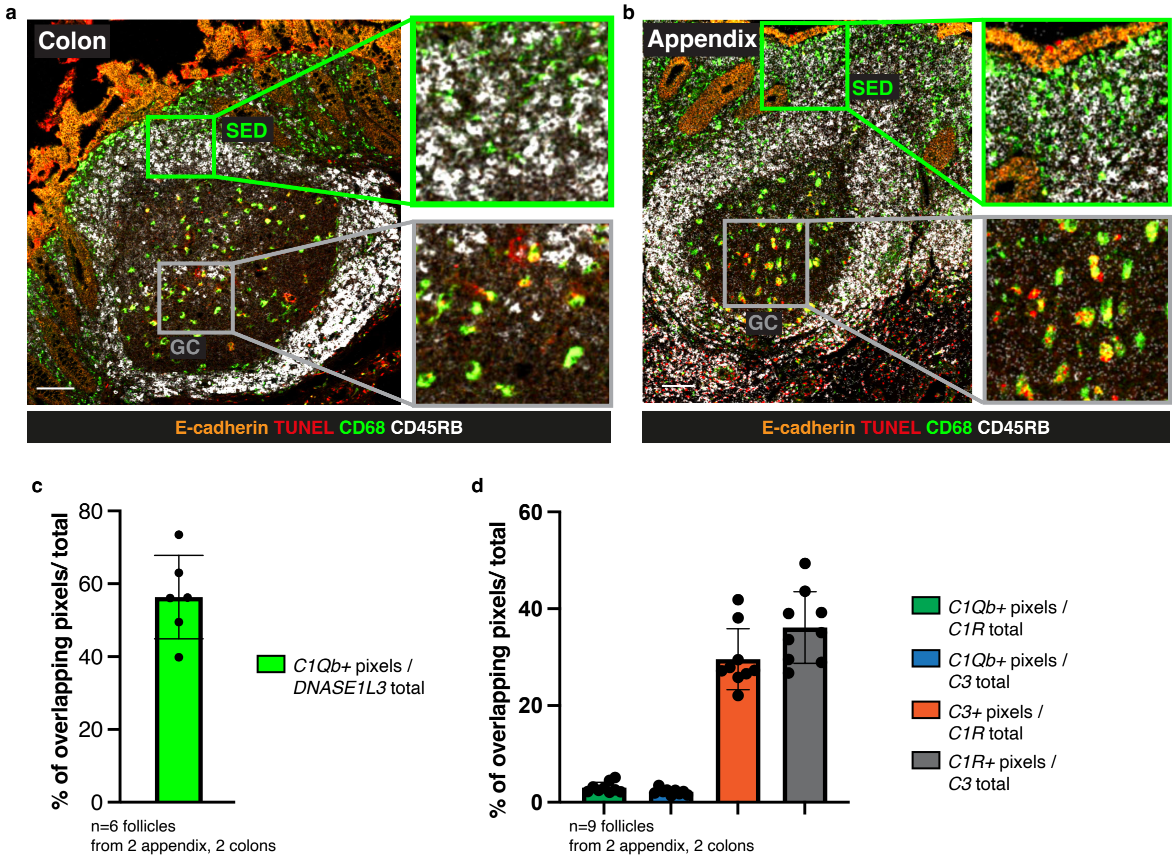

Supplementary Figure 6: Distribution of TUNEL positive apoptotic debris in appendix and colon and quantification of DNASE1L3 and complement component co-expression.

a, b IMC-derived image showing distribution of CD68 (green), cell apoptosis (TUNEL, red), CD45RB (white) and E-cadherin (orange) in colon and appendix follicles. Regions of subepithelial dome (SED) (green boxes) and germinal centre (GC) (grey boxes) are highlighted and enlarged. Scalebar=100μm. Findings are representative of 5 donors (ileum 3 donors, 5 ROI; appendix 3 donors, 5 ROI; colon 2 donors 6 ROI) in total. **c,d** Quantification of RNAscope analysis shown in Figure 7. **c** Percentage of *C1Qb*$^{+}$*DNASE1L3*$^{+}$ double positive pixels over total *DNASE1L3*$^{+}$ pixels, obtained from 6 follicles from 2 appendix and 2 colon donors. **d** Percentage of double positive pixels over total pixels for *C1Qb*$^{+}$*C1R*$^{+}$/total *C1R*$^{+}$*, C1Qb*$^{+}$*C3*$^{+}$/total *C3*$^{+}$*, C3*$^{+}$*C1R*$^{+}$/total *C1R*$^{+}$ and *C3*$^{+}$*C1R*$^{+}$/total *C3*$^{+}$, obtained from 9 follicles from 2 appendix and 2 colon donors. Source data are provided as a Source Data file.

Supplementary Table 1

Details of antibodies used for liquid Cytof
Indicates specificity, metal tag, clone, catalogue number, supplier and dilutions of antibodies used in liquid mass cytometry experiment

Marker	Metal Tag	Clone	Cat Number	Supplier	Dilution
BCMA	174Yb	19F2	357502	Biolegend	1:100
CCR10 (GPR-2)	166Er	314305	MAB3478	R&D Systems	1:100
CD10	158Gd	HI10a	3158011B	Fluidigm (now Standardbiotools)	1:100
CD138	150Nd	DL-101	3150012B	Fluidigm (now Standardbiotools)	1:100
CD180	176Yb	MHR73-11	312902	Biolegend	1:100
CD19	165Ho	HIB19	3165025B	Fluidigm (now Standardbiotools)	1:100
CD196 (CCR6)	141Pr	11A9	3141014A	Fluidigm (now Standardbiotools)	1:100
CD197 (CCR7)	175Lu	G043H7	353202	Biolegend	1:100
CD20	147Sm	2H7	3147001B	Fluidigm (now Standardbiotools)	1:100
CD21	152Sm	BL13	3152010B	Fluidigm (now Standardbiotools)	1:100
CD22	159Tb	HIB22	3159005B	Fluidigm (now Standardbiotools)	1:100
CD23	164Dy	EBVCS-5	3164018B	Fluidigm (now Standardbiotools)	1:100
CD24	169Tm	ML5	3169004B	Fluidigm (now Standardbiotools)	1:100
CD268 (BAFF-R)	155Gd	11C1	3155005B	Fluidigm (now Standardbiotools)	1:100
CD27	167Er	O323	3167002B	Fluidigm (now Standardbiotools)	1:100
CD29 (intergrin b1)	156Gd	TS2/16	3156007B	Fluidigm (now Standardbiotools)	1:100
CD38	154Sm	HIT2	303502	Biolegend	1:100
CD40	142Nd	5C3	3142010B	Fluidigm (now Standardbiotools)	1:100
CD45	89Y	HI30	3089003B	Fluidigm (now Standardbiotools)	1:100
CD45RB	145Nd	MEM-55	3145009B	Fluidigm (now Standardbiotools)	1:100
CD49d (a4)	151Eu	9F10	304302	Biolegend	1:100
CD5	143Nd	UCHT2	3143007B	Fluidigm (now Standardbiotools)	1:100
CD62L (L-selectin)	153Eu	DREG-56	3153004B	Fluidigm (now Standardbiotools)	1:100
CD69	144Nd	FN50	3144018B	Fluidigm (now Standardbiotools)	1:100
CD80 (B7-1)	161Dy	2D10.4	3161023B	Fluidigm (now Standardbiotools)	1:100
FcRL4	170Er	413D12	340202	Biolegend	1:100
HLA-DR	173Yb	L243	3173005B	Fluidigm (now Standardbiotools)	1:100
IgA	148Nd	Polyclonal	3148007B	Fluidigm (now Standardbiotools)	1:100
IgD	146Nd	IA6-2	3146005B	Fluidigm (now Standardbiotools)	1:100
IgG	171Yb	G18-145	555784	BD Biosciences	1:100
IgM	172Yb	MHM-88	3172004B	Fluidigm (now Standardbiotools)	1:100
Integrin b7	162Dy	FIB504	3162026B	Fluidigm (now Standardbiotools)	1:100
RANKL	149Sm	MIH24	347508	Biolegend	1:100
TACI	168Er	1A1	311902	Biolegend	1:100
CD3	160Gd	UCHT1	300402	Biolegend	1:100
CD14	160Gd	M5E2	3160001B	Fluidigm (now Standardbiotools)	1:100

<u>Supplementary Table 2</u>

Details of antibodies used in the panels of Imaging Mass Cytometry (IMC)
Indicates specificity, metal tag, clone, catalogue number and dilutions of antibodies used in imaging mass cytometry experiment

IMC PANEL 1						
	**Marker**	**Metal Tag**	**Cat number**	**Supplier**	**Clone**	**Dilution**
1	Lysozyme	141	860001	Biolegend	POLYCLONAL	200
2	CD11b	142	49420S	Cell Signaling Technology	D6X1N	1000
3	VIMENTIN	143	3143027D	Standardbiotools	D21H3	500
4	**FITC-TUNEL**	144	3144006B	Standardbiotools	FIT-22	150
5	CD38	145	ab226034	Abcam	EPR4106	100
6	BTLA	146	ab254287	Abcam	EPR22224-271	100
7	CD163	147	3147021D	Standardbiotools	EDHu-1	300
8	CD5	148	NBP2-34583	Novus Biologicals	C5/473 + CD5/54/F6	200
9	IgM	149	66484-1g	Fisher Scientific	2D10B10	100
10	CD11c	150	ab216655	Abcam	EP1347Y	400
11	CD31	151	3151025D	Standardbiotools	EPR3094	300
12	CD103	152	ab254201	Abcam	EPR22590-27	200
13	FCRL4	153	ab239754	Abcam	EPR21961	75
14	IgD	154	ab236778	Abcam	EPR6146	300
15	DNASE-I	155	ab113241	Abcam	polyclonal	200
16	CD45RB	156	310202	Biolegend	MEM-55	500
17	E-cad	158	3158029D	Standardbiotools	24E10	3000
18	CD68	159	3159035D	Standardbiotools	KP1	400
19	CD20	161	3161029D	Standardbiotools	H1	300
20	CD8	162	3162034D	Standardbiotools	C8/144B	400
21	LAMP3	163	353039	Biolegend	H5C6	500
22	CD1c	164	ab156708	Abcam	OTI2F4	500
23	CD74	166	3166018B	Standardbiotools	LN2	500
24	GzB	167	3167021D	Standardbiotools	EPR20129-217	300
25	Ki67	168	3168022D	Standardbiotools	B56	400
26	PDL1	169	MAB1561	RnD Systems	130021	150
27	CD3	170	3170019D	Standardbiotools	POLYCLONAL	400
28	CD27	171	3171024D	Standardbiotools	EPR8569	300
29	CD40	172	NBP2-34488	Novus Biologicals	CL1673	200
30	CD45RO	173	3173016D	Standardbiotools	UCHL1	500
31	NOX2	175	sc-130543	Santa Cruz	54.1	1000
32	CD83	176	ab275032	Abcam	EPR23809-19	300

IMC PANEL 2						
	**Marker**	**Metal Tag**	**Cat number**	**Supplier**	**Clone**	**Dilution**

1	Lysozyme	141	860001	Biolegend	POLYCLONAL	200
2	CD11b	142	49420S	Cell Signaling Technology	D6X1N	1000
3	VIMENTIN	143	3143027D	Standardbiotools	D21H3	500
4	CD38	145	ab226034	Abcam	EPR4106	100
5	BTLA	146	ab254287	Abcam	EPR22224-271	100
6	CD163	147	3147021D	Standardbiotools	EDHu-1	300
7	IgA	148	3148007B	Standardbiotools	polyclonal	200
8	IgM	149	66484-1-IG-20UL	Fisher Scientific	2D10B10	100
9	CD11c	150	ab216655	Abcam	EP1347Y	400
10	CD31	151	3151025D	Standardbiotools	EPR3094	300
11	CD103	152	ab254201	Abcam	EPR22590-27	200
12	FCRL4	153	ab239754	Abcam	EPR21961	75
13	IgD	154	ab236778	Abcam	EPR6146	300
14	DNASE-I	155	ab113241	Abcam	polyclonal	200
15	CD45RB	156	310202	Biolegend	MEM-55	250
16	E-cad	158	3158029D	Standardbiotools	24E10	3000
17	CD68	159	3159035D	Standardbiotools	KP1	400
18	CD8	162	3162034D	Standardbiotools	C8/144B	400
19	**Digoygenin - *DNASE1L3***	163	MAB7520	RnD Systems	611621	500
20	GzB	167	3167021D	Standardbiotools	EPR20129-217	300
21	Ki67	168	3168022D	Standardbiotools	B56	400
22	CD3	170	3170019D	Standardbiotools	POLYCLONAL	400
23	CD27	171	3171024D	Standardbiotools	EPR8569	300
24	CD40	172	NBP2-34488	Novus Biologicals	CL1673	200
25	CD45RO	173	3173016D	Standardbiotools	UCHL1	500
26	**FITC - *ITGAX***	174	3174006B	Standardbiotools	FIT-22	150
27	NOX2	175	sc-130543	Santa Cruz	54.1	750
28	CD83	176	ab275032	Abcam	EPR23809-19	300

IMC PANEL 3

	Marker	Metal Tag	Cat number	Supplier	Clone	Dilution
1	Lysozyme	141	860001	Biolegend	POLYCLONAL	200
2	CD11b	142	D6X1N	Cell Signaling Technology	D6X1N	1000
3	VIMENTIN	143	3143027D	Standardbiotools	D21H3	500
4	CD38	145	ab226034	Abcam	EPR4106	100
5	BTLA	146	ab254287	Abcam	EPR22224-271	100
6	CD163	147	3147021D	Standardbiotools	EDHu-1	300
7	IgA	148	3148007B	Standardbiotools	polyclonal	200
8	IgM	149	66484-1g	Fisher Scientific	2D10B10	100
9	CD11c	150	ab216655	Abcam	EP1347Y	400
10	CD31	151	3151025D	Standardbiotools	EPR3094	300

11	CD103	152	ab254201	Abcam	EPR22590-27	200
12	FCRL4	153	ab239754	Abcam	EPR21961	75
13	IgD	154	ab236778	Abcam	EPR6146	300
14	DNASE-I	155	ab113241	Abcam	polyclonal	200
15	CD45RB	156	310202	Biolegend	MEM-55	250
16	E-cad	158	3158029D	Standardbiotools	24E10	3000
17	CD68	159	3159035D	Standardbiotools	KP1	400
18	CD8	162	3162034D	Standardbiotools	C8/144B	400
19	**Digoygenin - *DNASE1L3***	163	MAB7520	RnD Systems	611621	500
20	**Biotin - Bacterial *16S***	166	409002	Biolegend	1D4-C5	150
21	GzB	167	3167021D	Standardbiotools	EPR20129-217	300
22	Ki67	168	3168022D	Standardbiotools	B56	400
23	CD3	170	3170019D	Standardbiotools	POLYCLONAL	400
24	CD27	171	3171024D	Standardbiotools	EPR8569	300
25	CD40	172	NBP2-34488	Novus Biologicals	CL1673	200
26	CD45RO	173	3173016D	Standardbiotools	UCHL1	500
27	**FITC - *ITGAX***	174	3174006B	Standardbiotools	FIT-22	150
28	NOX2	175	sc-130543	Santa Cruz	54.1	750
29	CD83	176	ab275032	Abcam	EPR23809-19	300

Supplementary Table 3

Differentially expressed genes identified in regions of images studied by spatial transcriptomics. Markers A lists the most differentially expressed gene between the SED and the rest of the follicle. Markers B lists differentially expressed genes in computationally defined subclusters. P values for differentially expressed genes were calculated using Wilcoxon tests with Bonferroni correction for multiple comparisons.

MARKERS A

gene	p_val	avg_log2FC_SED_vs_follicle	pct.1	pct.2	p_val_adj
DNASE1L3	4.84E-27	1.374169014	76.9%	5.7%	5.27E-23
IL22RA2	2.86E-19	0.700066011	51.3%	2.6%	3.11E-15
SOD3	7.30E-16	0.85274217	59.0%	7.3%	7.96E-12
CXCL14	1.09E-15	1.0500442	76.9%	17.1%	1.18E-11
PHLDA1	1.32E-15	0.586740465	46.2%	3.1%	1.44E-11
CLDN4	5.46E-14	1.281963837	66.7%	14.5%	5.96E-10
ATP1B1	7.09E-14	1.261457952	61.5%	10.9%	7.73E-10
CCL23	1.38E-13	1.307054818	51.3%	6.7%	1.50E-09
CCL20	4.73E-13	1.03823806	35.9%	2.1%	5.16E-09
ITGA3	2.16E-12	0.570089224	38.5%	3.1%	2.36E-08
PLAT	4.73E-12	0.4325857	35.9%	2.6%	5.16E-08
TSPAN8	9.14E-12	0.755412391	48.7%	7.3%	9.97E-08
ITGAX	1.05E-11	0.709325022	61.5%	13.5%	1.15E-07
PIGR	1.26E-11	0.835749099	64.1%	14.5%	1.38E-07
CTSE	1.84E-11	0.350998166	25.6%	0.5%	2.01E-07
TCF7L2	2.07E-11	0.674112289	51.3%	8.8%	2.26E-07
FABP1	2.24E-11	1.295082177	59.0%	13.5%	2.44E-07
SLC9A3	2.27E-11	0.88119435	56.4%	12.4%	2.47E-07
CEACAM1	4.42E-11	0.74762741	59.0%	13.0%	4.82E-07
COL6A2	4.72E-11	0.83312363	59.0%	14.5%	5.15E-07
SPINT1	5.10E-11	0.48505312	33.3%	2.6%	5.56E-07
TRIM31	5.90E-11	0.521179624	33.3%	2.6%	6.43E-07
SPINT2	7.48E-11	0.695979477	56.4%	11.9%	8.16E-07
BMP2	1.08E-10	0.439890501	30.8%	2.1%	1.17E-06
MUC13	1.23E-10	0.935495956	43.6%	6.7%	1.34E-06
HIST1H3H	1.26E-10	-1.286469696	20.5%	81.9%	1.37E-06
CDH1	1.44E-10	0.70219276	51.3%	9.8%	1.58E-06
MEF2B	1.66E-10	-1.453757518	28.2%	79.3%	1.80E-06
PLSCR1	1.77E-10	0.745456311	66.7%	18.7%	1.93E-06
MXD1	1.82E-10	0.392784692	28.2%	1.6%	1.99E-06
CCL15	2.03E-10	0.749059365	38.5%	4.7%	2.21E-06
ITM2C	2.81E-10	0.805394964	82.1%	26.9%	3.06E-06
FTH1	3.08E-10	0.861726854	97.4%	96.4%	3.35E-06
PHGR1	3.21E-10	1.533330674	66.7%	21.2%	3.50E-06
IL1B	3.80E-10	0.370869916	33.3%	3.1%	4.14E-06
ECE1	3.99E-10	0.608136128	64.1%	15.5%	4.35E-06
FERMT1	4.22E-10	0.499699896	33.3%	3.1%	4.61E-06
KLF4	5.60E-10	0.753253249	59.0%	15.0%	6.11E-06
LAD1	6.81E-10	0.439505114	41.0%	5.7%	7.43E-06
KRT8	7.87E-10	1.665685796	74.4%	34.7%	8.58E-06
ANP32E	8.35E-10	-1.102474102	79.5%	98.4%	9.11E-06
GALNT2	1.06E-09	0.52415294	61.5%	15.0%	1.16E-05
NR4A1	1.18E-09	0.708609088	46.2%	8.8%	1.29E-05
RNASET2	1.20E-09	0.669046083	79.5%	28.0%	1.31E-05
EPHA2	1.36E-09	0.412923454	28.2%	2.1%	1.49E-05
MMP14	1.38E-09	0.54015194	46.2%	8.8%	1.50E-05
KRT18	1.62E-09	0.54015194	41.0%	6.2%	1.76E-05
B2M	1.99E-09	0.900468984	100.0%	88.6%	2.17E-05

KRT20	2.38E-09	0.628982913	41.0%	6.7%	2.59E-05
PLS1	2.41E-09	0.420265429	25.6%	1.6%	2.63E-05
LMNA	2.52E-09	0.573841359	71.8%	21.8%	2.75E-05
EPCAM	2.58E-09	1.225666492	53.8%	15.0%	2.81E-05
CST3	2.59E-09	0.738771059	66.7%	20.2%	2.83E-05
TYROBP	2.71E-09	0.680098229	61.5%	17.6%	2.95E-05
HIST1H2AD	3.26E-09	-1.084266612	43.6%	85.0%	3.56E-05
TBC1D9	3.77E-09	0.51224515	59.0%	14.5%	4.12E-05
PDZK1IP1	3.99E-09	0.350998166	20.5%	0.5%	4.35E-05
IGFBP3	4.00E-09	0.697001337	59.0%	17.1%	4.37E-05
BHLHE40	4.14E-09	0.468598679	43.6%	7.8%	4.51E-05
CSF1R	4.20E-09	0.479235794	43.6%	7.8%	4.58E-05
TIMP1	4.54E-09	0.518558924	56.4%	14.0%	4.95E-05
LYZ	5.82E-09	1.433924629	82.1%	44.0%	6.34E-05
IGHG3	5.84E-09	0.813515345	61.5%	17.6%	6.37E-05
MICAL2	7.23E-09	0.594713011	46.2%	9.8%	7.88E-05
GPX4	1.01E-08	0.644089806	74.4%	29.0%	0.000109979
IFI27	1.03E-08	0.52944724	46.2%	9.8%	0.000111938
S100A6	1.11E-08	1.5470413	94.9%	76.7%	0.000121432
HIST1H4D	1.16E-08	-1.431793663	59.0%	93.8%	0.000126683
IGHA1	1.48E-08	1.995926124	94.9%	78.2%	0.000161656
TMEM59	1.58E-08	0.612793675	59.0%	18.7%	0.000172123
HIST1H3D	1.74E-08	-1.143606591	46.2%	89.1%	0.000190284
MYH14	1.84E-08	0.383923582	30.8%	3.6%	0.000201146
F3	2.05E-08	0.34285554	25.6%	2.1%	0.000223549
TNFRSF11A	2.12E-08	0.321554388	25.6%	2.1%	0.000231186
TCIM	2.24E-08	0.476240924	46.2%	9.8%	0.000243977
HIST1H1E	2.27E-08	-1.028948462	71.8%	99.0%	0.0002475
CDH17	2.55E-08	0.478535286	35.9%	5.7%	0.000277796
MMP9	2.84E-08	0.904295649	79.5%	39.4%	0.000309572
HLA-DQB2	2.87E-08	0.392784692	23.1%	1.6%	0.000312949
LGALS4	3.30E-08	0.812290127	64.1%	20.7%	0.000360138
HIST1H4C	3.36E-08	-1.102860957	51.3%	91.2%	0.000366655
LAMC2	3.77E-08	0.538646177	28.2%	3.1%	0.000410612
GPX2	3.83E-08	0.299712843	23.1%	1.6%	0.000417756
LITAF	3.84E-08	0.572151691	59.0%	18.1%	0.000418937
NFKBIA	4.36E-08	0.559820889	59.0%	18.1%	0.00047529
LAMP3	4.43E-08	0.441505018	35.9%	5.7%	0.000483426
CLU	4.67E-08	-1.68953858	69.2%	92.7%	0.000508848
PLAUR	4.85E-08	0.754513795	46.2%	10.9%	0.000528703
NCOA7	4.92E-08	0.628982913	59.0%	18.7%	0.000536872
ARRDC4	4.98E-08	0.421513543	46.2%	10.4%	0.000543226
GPRC5A	5.02E-08	0.538646177	30.8%	4.1%	0.000547619
BRI3	5.26E-08	0.553694786	59.0%	17.6%	0.000573397
IGKV4-1	5.40E-08	1.158458326	41.0%	8.3%	0.000589073
FXYD5	6.03E-08	0.428146635	46.2%	10.4%	0.000657977
SLC26A3	7.22E-08	0.903332632	56.4%	17.1%	0.000787331
MYO15B	7.58E-08	0.346947546	38.5%	6.7%	0.000826076
FOS	7.74E-08	0.909575691	53.8%	18.1%	0.000844359
TGFBI	8.08E-08	0.401914005	35.9%	6.2%	0.000881483
CYP3A5	9.42E-08	0.320994009	30.8%	4.1%	0.001027102
FXYD3	1.17E-07	0.932015387	53.8%	16.1%	0.001276926
MGAT1	1.28E-07	0.422047341	53.8%	14.5%	0.00139718
PLEC	1.38E-07	0.790340619	71.8%	32.1%	0.001500777
SEMA4C	1.80E-07	0.307054818	28.2%	3.6%	0.001961528
RALB	1.80E-07	0.307054818	28.2%	3.6%	0.001961528
ANPEP	1.87E-07	0.672339282	48.7%	12.4%	0.002043462
KLHL6	1.91E-07	-0.883083404	38.5%	79.3%	0.002079704
GNA11	2.09E-07	0.349283054	30.8%	4.7%	0.002276362
EIF4E3	2.09E-07	0.374169014	38.5%	7.8%	0.002283337
ETS2	2.25E-07	0.41598919	30.8%	4.7%	0.002450588
HIST1H1B	2.27E-07	-1.292101564	64.1%	88.6%	0.002470721
C1R	2.30E-07	0.682965986	71.8%	28.0%	0.002507552

CD44	2.36E-07	0.552167316	76.9%	30.6%	0.00257864
HIST1H4A	2.46E-07	-1.092116275	71.8%	95.9%	0.002687293
FOSB	2.75E-07	0.88559105	43.6%	11.9%	0.003000312
CLCA4	2.84E-07	0.812945748	25.6%	3.1%	0.003094766
HIST1H4F	2.89E-07	-0.957285785	43.6%	83.4%	0.003148487
SNRPD1	2.90E-07	-0.850091578	43.6%	81.3%	0.003161212
HIST1H3G	2.94E-07	-1.266680427	53.8%	82.4%	0.0032107
CDC42EP1	2.97E-07	0.37970012	38.5%	7.8%	0.003241487
CLDN7	3.24E-07	0.649028855	48.7%	13.5%	0.003534588
SKIL	3.53E-07	0.492086712	79.5%	32.6%	0.003846043
C1QC	3.67E-07	0.59361776	43.6%	10.9%	0.004006098
C3	3.71E-07	0.921401126	74.4%	39.9%	0.004041108
XDH	4.02E-07	0.314286388	25.6%	3.1%	0.004381076
GCNT3	4.46E-07	0.2699655	20.5%	1.6%	0.004868322
HIST1H4B	4.56E-07	-0.865495652	38.5%	77.2%	0.004973461
ENPP2	4.76E-07	0.396606403	30.8%	5.2%	0.005189716
IGFBP7	4.87E-07	0.614032572	89.7%	58.0%	0.005310526
CLDN3	5.12E-07	0.560811411	30.8%	5.2%	0.005578095
DSC2	5.61E-07	0.389516979	30.8%	5.2%	0.006120209
ZFP36	5.94E-07	0.525335823	59.0%	19.7%	0.006475586
RCAN1	6.15E-07	0.369639356	28.2%	4.1%	0.006711318
BANK1	6.47E-07	0.49382333	61.5%	22.3%	0.007053757
CORO1A	6.78E-07	-0.697945863	74.4%	99.0%	0.007388795
HIST1H4H	7.12E-07	-0.933367018	43.6%	80.8%	0.00776428
LCN2	7.18E-07	0.565122971	15.4%	0.5%	0.007829189
C1QA	7.30E-07	0.285575091	28.2%	4.1%	0.007963463
AGAP1	7.30E-07	0.292699525	28.2%	4.1%	0.007963463
AQP8	8.09E-07	0.387974814	30.8%	5.2%	0.00882015
POU2AF1	8.96E-07	-0.815173441	69.2%	89.1%	0.009774381
ID1	9.47E-07	0.400164223	33.3%	6.2%	0.010325574
LGALS2	9.66E-07	0.368455363	30.8%	5.2%	0.010532805
COL3A1	9.70E-07	0.6066151	82.1%	38.9%	0.010582144
SERF2	9.84E-07	-0.702697865	97.4%	99.5%	0.010730337
HNF4A	1.03E-06	0.262660699	23.1%	2.6%	0.011188568
TSPAN1	1.03E-06	0.690796187	46.2%	13.5%	0.011204753
TNFRSF21	1.03E-06	0.503082536	38.5%	9.3%	0.011243496
LSR	1.03E-06	0.668899379	41.0%	10.9%	0.01124982
DDR1	1.31E-06	0.489918876	43.6%	11.9%	0.014286452
RNF130	1.31E-06	0.363638347	25.6%	3.6%	0.0143255
ICAM1	1.36E-06	0.519599454	61.5%	22.8%	0.014857333
HIST1H2BD	1.40E-06	-0.760059377	43.6%	79.8%	0.015293554
RAB5A	1.65E-06	0.387673279	69.2%	26.4%	0.017969031
CEACAM7	1.69E-06	0.362549931	25.6%	3.6%	0.018469445
HKDC1	1.71E-06	0.292699525	25.6%	3.6%	0.018596756
MALL	1.86E-06	0.523546638	33.3%	7.3%	0.020274252
DHCR7	1.89E-06	0.292699525	28.2%	4.7%	0.020630649
C1orf116	2.11E-06	0.2699655	20.5%	2.1%	0.023062267
DLL1	2.11E-06	0.2699655	20.5%	2.1%	0.023062267
CD177	2.11E-06	0.2699655	20.5%	2.1%	0.023062267

MARKERS B

subcluster	gene	p_val	avg_log2FC	pct.1	pct.2	p_val_adj
B cell/T cell	FCGRT	1.74E-06	0.508365537	85.3%	58.7%	0.026199572
B cell/T cell	ANP32E	4.32E-06	0.39826892	100.0%	93.4%	0.065245481
B cell/T cell	GOLPH3	4.52E-06	0.565589631	89.7%	65.9%	0.068183356
B cell/T cell	G3BP2	6.47E-06	0.505694817	89.7%	67.7%	0.097679051
B cell/T cell	ARGLU1	1.39E-05	0.462019923	97.1%	82.0%	0.209645692
B cell/T cell	CNBP	1.68E-05	0.51165142	94.1%	88.0%	0.254041402
B cell/T cell	SNRNP70	9.03E-05	0.470154161	94.1%	95.2%	1
B cell/T cell	GABARAP	9.22E-05	0.478988068	97.1%	94.0%	1
B cell/T cell	BANF1	0.000179661	0.461125836	94.1%	75.4%	1
B cell/T cell	SMCHD1	0.000236388	0.388359653	85.3%	61.7%	1

B cell/T cell	PRRC2C	0.00026215	0.424846103	98.5%	88.6%	1
B cell/T cell	CCDC91	0.000272271	0.447108117	57.4%	34.7%	1
B cell/T cell	HSPA5	0.000340258	0.40169075	86.8%	73.7%	1
B cell/T cell	CIITA	0.000451357	0.4198728	88.2%	68.3%	1
B cell/T cell	MRFAP1L1	0.000757434	0.535429115	64.7%	50.9%	1
B cell/T cell	HNRNPDL	0.000781041	0.344424985	98.5%	98.2%	1
B cell/T cell	TRBC1	0.001164545	0.475790872	64.7%	46.1%	1
B cell/T cell	PTGDS	0.001266905	0.517819021	85.3%	68.9%	1
B cell/T cell	RBM42	0.001862604	0.443467404	48.5%	32.3%	1
B cell/T cell	IKBKE	0.002244257	0.366944443	42.6%	25.7%	1
B cell/T cell	EEF1D	0.002395846	0.369770487	67.6%	53.9%	1
B cell/T cell	C4orf3	0.002408081	0.345780912	92.6%	80.8%	1
B cell/T cell	MOB3B	0.002424212	0.296241451	27.9%	12.6%	1
B cell/T cell	PNISR	0.003387616	0.346867524	79.4%	61.1%	1
B cell/T cell	COPS9	0.004256396	0.52477301	54.4%	40.1%	1
B cell/T cell	TRBJ2-2	0.006811831	0.280974695	25.0%	11.4%	1
B cell/T cell	LUC7L3	0.00743797	0.317721179	88.2%	70.7%	1
B cell/T cell	TMEM230	0.007817293	0.432876143	64.7%	54.5%	1
B cell/T cell	GTF3C6	0.008850565	0.360371789	77.9%	64.7%	1
B cell/T cell	FUS	0.009444251	0.267178986	80.9%	68.9%	1
GC 1	HIST1H4D	2.57E-16	1.193498155	100.0%	86.7%	3.89E-12
GC 1	HIST1H4A	1.69E-14	1.150585211	100.0%	91.0%	2.55E-10
GC 1	PFN1	1.31E-13	0.652573407	100.0%	100.0%	1.98E-09
GC 1	ANP32E	1.84E-13	0.964927233	100.0%	94.1%	2.78E-09
GC 1	HMGA1	5.83E-13	0.920432743	100.0%	84.6%	8.80E-09
GC 1	ARPC2	1.93E-12	0.692644706	100.0%	98.4%	2.91E-08
GC 1	SERF2	2.15E-12	0.710300238	100.0%	100.0%	3.24E-08
GC 1	IGLJ1	3.37E-12	0.537209369	55.3%	10.1%	5.08E-08
GC 1	SERPINA9	1.04E-11	0.616919267	74.5%	19.7%	1.58E-07
GC 1	BIK	1.15E-11	0.711112167	78.7%	24.5%	1.74E-07
GC 1	MEF2B	1.45E-11	0.980332967	95.7%	65.4%	2.19E-07
GC 1	HIST1H4F	3.66E-11	0.879705766	97.9%	70.7%	5.52E-07
GC 1	SNRPD1	7.60E-11	0.801066551	97.9%	69.7%	1.15E-06
GC 1	SPC24	1.11E-10	0.636529643	61.7%	16.5%	1.67E-06
GC 1	HIST1H3D	1.15E-10	0.856010728	100.0%	81.4%	1.73E-06
GC 1	PABPC1	2.00E-10	0.521844226	100.0%	100.0%	3.02E-06
GC 1	HIST1H2BN	2.70E-10	0.888601075	97.9%	70.2%	4.07E-06
GC 1	ATP5IF1	1.57E-09	0.71302754	97.9%	85.1%	2.37E-05
GC 1	HIST1H4B	1.71E-09	0.786695451	95.7%	62.8%	2.57E-05
GC 1	CDCA7	2.49E-09	0.797981369	91.5%	49.5%	3.76E-05
GC 1	HIST1H1B	3.06E-09	0.752072487	100.0%	84.6%	4.62E-05
GC 1	HIST1H4H	4.33E-09	0.676730487	95.7%	70.7%	6.54E-05
GC 1	SLBP	7.02E-09	0.651545611	66.0%	24.5%	0.000105892
GC 1	SAPCD2	9.38E-09	0.393663848	38.3%	6.4%	0.000141574
GC 1	CCDC88A	1.01E-08	0.845181891	72.3%	33.0%	0.000151762
GC 1	NDUFA7	1.12E-08	0.526747887	61.7%	19.1%	0.000169738
GC 1	HIST1H3G	1.28E-08	0.856496176	95.7%	74.5%	0.00019256
GC 1	WEE1	2.02E-08	0.543485865	68.1%	26.1%	0.000304866
GC 1	POU2AF1	3.27E-08	0.614762514	100.0%	80.9%	0.000492981
GC 1	HIST1H1E	3.39E-08	0.606498585	100.0%	96.3%	0.000511697
GC 1	CD79A	3.54E-08	0.623279432	100.0%	88.8%	0.000534292
GC 1	PFAS	3.63E-08	0.447458977	51.1%	13.8%	0.000548258
GC 1	HAUS8	3.71E-08	0.439912168	55.3%	16.0%	0.000559742
GC 1	CHCHD2	4.75E-08	0.545660851	97.9%	95.7%	0.000716204
GC 1	HNRNPA3	5.37E-08	0.569365646	100.0%	99.5%	0.000810321
GC 1	BPNT1	6.87E-08	0.546542282	61.7%	22.9%	0.001037254
GC 1	HIST1H2AD	6.98E-08	0.676834071	97.9%	75.0%	0.001052787
GC 1	CENPM	9.19E-08	0.552541023	61.7%	22.3%	0.001386608
GC 1	PRC1	1.70E-07	0.485426827	48.9%	14.4%	0.002564114
GC 1	EEF2	1.71E-07	0.371293263	100.0%	100.0%	0.00257986
GC 1	CDC6	1.83E-07	0.3205199	36.2%	6.9%	0.002768865
GC 1	FAM241A	2.06E-07	0.415037499	44.7%	11.7%	0.003109895
GC 1	ODC1	2.20E-07	0.637429921	83.0%	49.5%	0.003321606

GC 1	NUSAP1	2.48E-07	0.505235308	61.7%	22.3%	0.003747684
GC 1	METAP2	2.56E-07	0.674229839	87.2%	53.2%	0.003860082
GC 1	HIST1H4C	3.07E-07	0.533179959	100.0%	83.0%	0.004640972
GC 1	MXD3	3.18E-07	0.366363653	42.6%	10.6%	0.004796405
GC 1	ERH	3.18E-07	0.678071905	78.7%	48.9%	0.004806907
GC 1	SEL1L3	3.26E-07	0.644771896	95.7%	77.7%	0.004921269
GC 1	IMP4	3.45E-07	0.49336586	55.3%	18.6%	0.005207182
GC 1	HIST2H2AB	3.64E-07	0.711812117	89.4%	62.2%	0.005494159
GC 1	NSD2	4.68E-07	0.33137957	42.6%	10.6%	0.007064047
GC 1	PLK1	4.78E-07	0.509364882	48.9%	15.4%	0.007221696
GC 1	SMCO4	4.83E-07	0.365649472	40.4%	10.1%	0.007287258
GC 1	PSMD10	5.00E-07	0.474908955	55.3%	19.7%	0.007552591
GC 1	TMED8	5.14E-07	0.519734878	72.3%	33.0%	0.00775256
GC 1	CDCA4	5.54E-07	0.401165325	38.3%	9.0%	0.008355401
GC 1	BCAS4	5.76E-07	0.619005964	87.2%	50.0%	0.008695604
GC 1	COX7B	6.23E-07	0.350907162	38.3%	9.0%	0.009401887
GC 1	AURKB	6.86E-07	0.375509135	48.9%	14.4%	0.010347815
GC 1	HIST1H3I	7.53E-07	0.569071128	95.7%	59.6%	0.011362366
GC 1	CDC20	8.21E-07	0.76611194	66.0%	33.0%	0.012396211
GC 1	UCHL1	9.79E-07	0.505235308	44.7%	13.8%	0.014777253
GC 1	FDX1	1.02E-06	0.387816031	42.6%	11.7%	0.015458025
GC 1	H2AFX	1.05E-06	0.526068812	70.2%	30.3%	0.015786276
GC 1	HIST1H2BO	1.33E-06	0.571627862	91.5%	59.0%	0.020014826
GC 1	BRCA1	1.33E-06	0.358730058	38.3%	9.6%	0.020078309
GC 1	STMN1	1.51E-06	0.442004547	40.4%	11.2%	0.022790896
GC 1	DMAC2L	1.57E-06	0.284838776	27.7%	4.8%	0.02369873
GC 1	DDB2	1.69E-06	0.282730207	23.4%	3.2%	0.025562988
GC 1	RAE1	1.83E-06	0.31757169	29.8%	5.9%	0.027600635
GC 1	PPP6C	1.84E-06	0.277533976	27.7%	4.8%	0.027779692
GC 1	CENPL	1.87E-06	0.292180751	23.4%	3.2%	0.028225955
GC 1	KLHL6	1.88E-06	0.593374741	93.6%	66.0%	0.028310005
GC 1	CPSF4	2.53E-06	0.373217324	40.4%	11.7%	0.038225627
GC 1	DONSON	2.79E-06	0.33137957	40.4%	11.2%	0.042100128
GC 1	BANF1	3.18E-06	0.512884823	93.6%	77.7%	0.047990421
GC 1	TCL1A	3.31E-06	0.580461108	89.4%	58.5%	0.049997747
GC 1	PRKRA	3.37E-06	0.304854582	31.9%	6.9%	0.050901389
GC 1	NCAPH	3.52E-06	0.483601786	46.8%	15.4%	0.053112297
GC 1	ASF1B	3.58E-06	0.402600396	51.1%	18.1%	0.054035371
GC 1	ARHGEF9	3.61E-06	0.26059123	25.5%	4.3%	0.054546848
GC 1	HDGF	3.65E-06	0.529042128	85.1%	59.0%	0.055155174
GC 1	MCM6	4.02E-06	0.409984272	74.5%	32.4%	0.060710656
GC 1	DTX1	4.08E-06	0.532321021	72.3%	37.8%	0.061556105
GC 1	MELK	4.08E-06	0.442004547	38.3%	11.2%	0.061577124
GC 1	PGAM1	4.89E-06	0.581934808	72.3%	48.9%	0.073873266
GC 1	NCAPD2	4.94E-06	0.436099115	66.0%	28.7%	0.074508172
GC 1	POLDIP2	5.39E-06	0.362570079	48.9%	17.0%	0.081343942
GC 1	SGPP1	5.69E-06	0.621488377	72.3%	44.1%	0.085909486
GC 1	HIST1H2BJ	5.74E-06	0.35614381	57.4%	21.3%	0.086685254
GC 1	OGG1	5.77E-06	0.403212064	61.7%	27.1%	0.087151022
GC 1	C16orf87	6.03E-06	0.250650681	23.4%	3.7%	0.090952408
GC 1	CDC25B	6.25E-06	0.363276072	46.8%	15.4%	0.094287266
GC 1	ARPC3	7.53E-06	0.463428983	97.9%	86.7%	0.113649936
GC 1	GZF1	7.80E-06	0.353636955	40.4%	12.2%	0.117793863
GC 1	PPM1G	7.99E-06	0.580869746	83.0%	56.4%	0.120599028
GC 1	TPX2	8.14E-06	0.524420959	68.1%	34.0%	0.12290443
GC 1	SRSF3	1.04E-05	0.400661771	95.7%	93.1%	0.156894493
GC 1	EHMT2	1.06E-05	0.270265975	27.7%	5.9%	0.160081469
GC 1	OAZ1	1.15E-05	0.441269041	95.7%	96.8%	0.172965956
GC 1	ENO1	1.16E-05	0.609880133	91.5%	88.8%	0.17473397
GC 1	PATZ1	1.30E-05	0.343954401	36.2%	10.6%	0.196155719
GC 1	CENPF	1.33E-05	0.258311996	29.8%	6.9%	0.200894709
GC 1	SMC4	1.39E-05	0.36705747	38.3%	11.7%	0.209592324
GC 1	CPSF3	1.41E-05	0.255838904	27.7%	5.9%	0.212089869

GC 1	RFTN1	1.43E-05	0.546949175	63.8%	30.9%	0.215780869
GC 1	HIST1H4I	1.47E-05	0.623624121	91.5%	69.1%	0.222252246
GC 1	CPNE5	1.61E-05	0.415037499	61.7%	27.1%	0.243285645
GC 1	POLQ	1.62E-05	0.32483969	25.5%	5.3%	0.243855974
GC 1	NEIL1	1.62E-05	0.46457696	46.8%	17.6%	0.244689359
GC 1	POLR2L	1.68E-05	0.488865911	89.4%	71.3%	0.254259957
GC 1	DNAJC9	1.74E-05	0.309684499	38.3%	11.7%	0.263143284
GC 1	PPP2R3B	1.76E-05	0.327574658	31.9%	8.5%	0.266370332
GC 1	HIST1H2AB	1.84E-05	0.59946207	70.2%	38.8%	0.27763702
GC 1	HIST1H2AE	2.04E-05	0.533600697	89.4%	66.0%	0.307614134
GC 1	PRKCD	2.07E-05	0.378968245	57.4%	25.0%	0.3128574
GC 1	FCGR2B	2.21E-05	0.394859617	40.4%	13.8%	0.333424284
GC 1	SMARCD2	2.28E-05	0.389274403	46.8%	18.1%	0.343922611
GC 1	PPP1CC	2.34E-05	0.491388385	63.8%	33.5%	0.35313849
GC 1	SMC1A	2.40E-05	0.395137942	40.4%	13.8%	0.362569148
GC 1	CLU	2.42E-05	0.360634355	100.0%	85.6%	0.36528267
GC 1	TAF12	2.47E-05	0.302927133	40.4%	13.3%	0.37278446
GC 1	RASGRP3	2.49E-05	0.472752997	53.2%	23.4%	0.375632957
GC 1	NIBAN1	2.50E-05	0.288860393	29.8%	7.4%	0.376773142
GC 1	TACC3	2.71E-05	0.362570079	44.7%	16.5%	0.408719252
GC 1	NDUFA2	2.80E-05	0.418920934	93.6%	62.2%	0.423049649
GC 1	ACY3	2.84E-05	0.26540142	29.8%	7.4%	0.427954118
GC 1	HIST1H2BB	2.99E-05	0.559958495	80.9%	52.1%	0.451914954
GC 1	OSBPL9	3.06E-05	0.38332864	48.9%	20.2%	0.461734352
GC 1	FAM83D	3.16E-05	0.250650681	21.3%	3.7%	0.477220909
GC 1	GRHPR	3.20E-05	0.403495877	57.4%	25.0%	0.482459714
GC 1	CHEK1	3.24E-05	0.324622204	34.0%	9.6%	0.488545016
GC 1	NDUFS7	3.36E-05	0.306513043	34.0%	10.1%	0.507769444
GC 1	ATP5F1B	3.63E-05	0.41814341	95.7%	77.1%	0.547829967
GC 1	ACTR3	3.84E-05	0.433261258	100.0%	93.1%	0.579655658
GC 1	CENPN	3.85E-05	0.327574658	29.8%	8.0%	0.581487137
GC 1	UBE2C	3.86E-05	0.415037499	53.2%	22.3%	0.581941776
GC 1	SYPL1	4.07E-05	0.359542387	36.2%	11.7%	0.614118509
GC 1	PSRC1	4.11E-05	0.26059123	23.4%	4.8%	0.619681342
GC 1	COMMD4	4.32E-05	0.327951883	51.1%	20.2%	0.652245484
GC 1	ASB13	4.34E-05	0.287658193	36.2%	11.2%	0.654998994
GC 1	MARCKSL1	4.42E-05	0.418184055	93.6%	64.4%	0.667629037
GC 1	TRAPPC1	4.51E-05	0.378511623	53.2%	22.9%	0.680172264
GC 1	LSM10	4.78E-05	0.454721201	55.3%	25.0%	0.721165038
GC 1	HIST1H2BI	4.99E-05	0.430736186	93.6%	63.8%	0.75280511
GC 1	PAN2	5.01E-05	0.26540142	29.8%	8.0%	0.756287516
GC 1	DEK	5.10E-05	0.453640339	95.7%	81.9%	0.769676096
GC 1	NCEH1	5.20E-05	0.269920791	31.9%	9.0%	0.784298991
GC 1	HIST1H2AI	5.82E-05	0.458809901	87.2%	54.8%	0.879121738
GC 1	CUX1	5.91E-05	0.31410859	40.4%	13.8%	0.892605445
GC 1	JPT1	6.33E-05	0.56751353	68.1%	42.6%	0.955216633
GC 1	KLHL5	6.44E-05	0.428584031	78.7%	44.1%	0.971467879
GC 1	USP22	6.47E-05	0.53518853	87.2%	67.0%	0.976966888
GC 1	SNRNP25	6.67E-05	0.469485283	74.5%	40.4%	1
GC 1	IGHA2	6.74E-05	0.342392197	40.4%	14.4%	1
GC 1	STOML2	6.87E-05	0.315008681	34.0%	10.6%	1
GC 1	PSMB1	7.44E-05	0.419538892	76.6%	47.3%	1
GC 1	SYAP1	7.56E-05	0.37455843	53.2%	22.9%	1
GC 1	COX17	7.93E-05	0.540568381	68.1%	43.1%	1
GC 1	C1orf52	8.60E-05	0.293046975	40.4%	14.4%	1
GC 1	DCK	8.70E-05	0.420162531	68.1%	35.6%	1
GC 1	PLEKHJ1	8.75E-05	0.26529038	36.2%	11.7%	1
GC 1	CDK4	9.23E-05	0.49426419	83.0%	59.0%	1
GC 1	GPR89B	9.40E-05	0.272525855	27.7%	7.4%	1
GC 1	THAP4	9.49E-05	0.290779396	29.8%	8.5%	1
GC 1	ANAPC11	9.50E-05	0.294447358	36.2%	12.2%	1
GC 1	MTA3	9.55E-05	0.500967678	59.6%	30.9%	1
GC 1	CENPP	9.57E-05	0.287658193	36.2%	12.2%	1

GC 1	CENPA	9.61E-05	0.253286429	23.4%	5.3%	1
GC 1	SPAG5	9.61E-05	0.253286429	23.4%	5.3%	1
GC 1	MDM2	0.00010273	0.32311501	48.9%	20.2%	1
GC 1	TUT7	0.000104059	0.430979043	61.7%	34.0%	1
GC 1	PRPS1	0.000105827	0.342392197	42.6%	16.5%	1
GC 1	GON4L	0.000109155	0.263034406	23.4%	5.3%	1
GC 1	PRDX6	0.000110256	0.478165478	70.2%	43.1%	1
GC 1	CCT3	0.000116247	0.42946457	85.1%	64.9%	1
GC 1	GID8	0.000120803	0.286562941	42.6%	16.0%	1
GC 1	HK1	0.000125378	0.299560282	44.7%	17.6%	1
GC 1	DNPEP	0.000126865	0.335965928	44.7%	18.6%	1
GC 1	KPNA2	0.000126967	0.358047521	66.0%	33.0%	1
GC 1	MYBL1	0.000126982	0.35310975	46.8%	18.6%	1
GC 1	AFF2	0.000129026	0.420575683	55.3%	27.7%	1
GC 1	GSTP1	0.000137722	0.42392933	97.9%	92.6%	1
GC 1	S100Z	0.000138916	0.276209795	36.2%	12.2%	1
GC 1	ZNRD1	0.0001515	0.313499473	29.8%	9.0%	1
GC 1	SMPD4	0.000154818	0.297904866	38.3%	13.8%	1
GC 1	SET	0.000156694	0.374218595	93.6%	78.2%	1
GC 1	SNRPB	0.000159174	0.372512883	46.8%	19.7%	1
GC 1	MYBL2	0.000163719	0.352976531	63.8%	31.4%	1
GC 1	RBM38	0.000180664	0.415037499	57.4%	30.3%	1
GC 1	NANS	0.000182914	0.420706249	55.3%	26.6%	1
GC 1	DDX39A	0.00018884	0.359542387	59.6%	29.3%	1
GC 1	S1PR2	0.000190969	0.32311501	48.9%	20.7%	1
GC 1	LIG1	0.00019385	0.36184456	38.3%	14.4%	1
GC 1	MCM7	0.000198185	0.565317972	78.7%	65.4%	1
GC 1	PXMP2	0.000201326	0.258311996	27.7%	8.0%	1
GC 1	PDZD11	0.000201326	0.258311996	27.7%	8.0%	1
GC 1	BCL7C	0.000205227	0.272815427	53.2%	23.4%	1
GC 1	HIST1H2BD	0.000207323	0.428458015	78.7%	69.1%	1
GC 1	PRELID3B	0.000209048	0.442310429	57.4%	31.4%	1
GC 1	DNMT1	0.000209197	0.485426827	72.3%	51.6%	1
GC 1	ARPC4	0.000210969	0.368802033	97.9%	85.1%	1
GC 1	RFC1	0.000211257	0.309104055	42.6%	17.0%	1
GC 1	MCM2	0.000212618	0.304537958	36.2%	12.8%	1
GC 1	CR2	0.000214366	0.368306449	85.1%	64.4%	1
GC 1	KIF11	0.000223556	0.276209795	36.2%	12.8%	1
GC 1	UBE2E2	0.000238108	0.332936419	44.7%	19.1%	1
GC 1	DDX1	0.000238349	0.290779396	29.8%	9.6%	1
GC 1	COTL1	0.000242462	0.394476177	97.9%	93.1%	1
GC 1	PMVK	0.000243996	0.359895945	46.8%	20.7%	1
GC 1	ROMO1	0.000246689	0.584962501	72.3%	58.5%	1
GC 1	VDAC1	0.000261901	0.435005916	91.5%	85.6%	1
GC 1	LMO2	0.000262242	0.343778816	44.7%	18.6%	1
GC 1	RCC1	0.000273435	0.267695783	29.8%	9.6%	1
GC 1	POLD1	0.0002976	0.306103128	38.3%	14.9%	1
GC 1	LY86	0.000303965	0.344115713	61.7%	31.9%	1
GC 1	GATD1	0.000304364	0.295455884	57.4%	28.2%	1
GC 1	EHMT1	0.000309521	0.31748219	55.3%	26.6%	1
GC 1	PDCD5	0.000315204	0.348695004	59.6%	30.9%	1
GC 1	TRIM41	0.000316536	0.256180735	31.9%	10.6%	1
GC 1	RAD21	0.000320149	0.25649156	38.3%	14.4%	1
GC 1	FCRLB	0.000320528	0.267480311	36.2%	13.3%	1
GC 1	HNRNPD	0.000327444	0.372809264	72.3%	45.2%	1
GC 1	PTK2B	0.000328367	0.327951883	48.9%	21.8%	1
GC 1	SNAP47	0.000331691	0.288345494	40.4%	16.0%	1
GC 1	MSH2	0.000332036	0.263034406	48.9%	21.3%	1
GC 1	ATP5F1A	0.000332416	0.428212888	74.5%	47.9%	1
GC 1	EIF3A	0.000342059	0.381731413	91.5%	80.3%	1
GC 1	LPP	0.000346536	0.285560975	44.7%	18.6%	1
GC 1	NCL	0.000359766	0.378511623	91.5%	84.6%	1
GC 1	CCDC69	0.000363511	0.441371647	80.9%	59.6%	1

GC 1	CDT1	0.000369291	0.337996464	48.9%	21.8%	1
GC 1	SEM1	0.000370567	0.385058342	85.1%	60.6%	1
GC 1	HNRNPUL1	0.000376939	0.373065229	97.9%	96.8%	1
GC 1	TUBA4A	0.000378153	0.389758091	87.2%	69.1%	1
GC 1	CBX5	0.000386635	0.472487771	68.1%	45.7%	1
GC 1	CCNB2	0.000400074	0.267480311	34.0%	12.2%	1
GC 1	TXNDC17	0.000405397	0.315501826	40.4%	16.5%	1
GC 1	TMEM60	0.00041414	0.255838904	23.4%	6.4%	1
GC 1	ARPC1B	0.000415705	0.401981346	95.7%	95.2%	1
GC 1	NDUFB10	0.000438233	0.359895945	44.7%	19.7%	1
GC 1	SAE1	0.000440443	0.260709353	27.7%	8.5%	1
GC 1	NAA38	0.000448392	0.386008451	55.3%	29.3%	1
GC 1	NONO	0.000461655	0.332727146	95.7%	87.8%	1
GC 1	CAP1	0.000468534	0.419903254	63.8%	36.7%	1
GC 1	KRT10	0.000469973	0.335038177	48.9%	23.9%	1
GC 1	PIK3CG	0.000477997	0.263034406	36.2%	13.8%	1
GC 1	SPI1	0.000480243	0.423574181	74.5%	55.3%	1
GC 1	SREBF2	0.00048185	0.390584456	46.8%	22.9%	1
GC 1	DBI	0.000488065	0.419118676	80.9%	55.3%	1
GC 1	TIMM8B	0.00050628	0.398549376	55.3%	31.4%	1
GC 1	ILF2	0.000516485	0.32311501	48.9%	22.9%	1
GC 1	NOM1	0.00052244	0.376043368	40.4%	17.6%	1
GC 1	AHSA1	0.000540255	0.454208097	70.2%	47.3%	1
GC 1	HLA-DOA	0.000548202	0.309684499	61.7%	31.9%	1
GC 1	C11orf98	0.000563326	0.276383959	53.2%	24.5%	1
GC 1	RPA1	0.000569515	0.251257238	27.7%	9.0%	1
GC 1	AFMID	0.000569515	0.251257238	27.7%	9.0%	1
GC 1	GMPS	0.000574484	0.283792966	42.6%	18.1%	1
GC 1	RAB30	0.000574555	0.258533014	34.0%	12.8%	1
GC 1	C21orf58	0.000574555	0.258533014	34.0%	12.8%	1
GC 1	NAA20	0.0005797	0.299560282	38.3%	16.0%	1
GC 1	BLOC1S6	0.000621359	0.281975969	40.4%	17.0%	1
GC 1	KCTD20	0.000650217	0.25649156	36.2%	14.4%	1
GC 1	SNX3	0.000654986	0.409875794	59.6%	33.5%	1
GC 1	GPS1	0.000719738	0.263034406	36.2%	14.4%	1
GC 1	TXN2	0.000734352	0.334225454	55.3%	29.8%	1
GC 1	SWAP70	0.000751262	0.364764293	66.0%	36.7%	1
GC 1	MRTO4	0.000766813	0.271673324	36.2%	14.4%	1
GC 1	TIMM10	0.000786764	0.290079229	42.6%	19.1%	1
GC 1	INCENP	0.000811633	0.316633795	57.4%	28.7%	1
GC 1	PDS5A	0.000839156	0.415037499	63.8%	41.5%	1
GC 1	SIVA1	0.000841826	0.332789088	83.0%	66.0%	1
GC 1	LMNB2	0.000851732	0.308586558	42.6%	19.1%	1
GC 1	SOD1	0.000855861	0.36705747	87.2%	67.0%	1
GC 1	USP6NL	0.000871338	0.251061764	48.9%	22.9%	1
GC 1	EIF4E2	0.000886532	0.268017813	63.8%	31.9%	1
GC 1	LUC7L2	0.000893291	0.283792966	61.7%	33.5%	1
GC 1	LPCAT1	0.000928645	0.35379987	80.9%	62.8%	1
GC 1	PHF19	0.000957208	0.308950241	57.4%	29.3%	1
GC 1	RNF34	0.000981007	0.25180715	31.9%	11.7%	1
GC 1	HDAC1	0.00100284	0.410283969	63.8%	39.4%	1
GC 1	COX8A	0.001032859	0.415037499	87.2%	69.1%	1
GC 1	GTSE1	0.001076079	0.35614381	38.3%	17.0%	1
GC 1	DNAJC7	0.001100728	0.383673328	59.6%	35.1%	1
GC 1	LIMD2	0.001107248	0.331807826	97.9%	88.3%	1
GC 1	TXNDC15	0.001127673	0.290079229	40.4%	18.1%	1
GC 1	EIF2D	0.001164884	0.334000927	44.7%	21.8%	1
GC 1	C1orf35	0.001170245	0.258311996	25.5%	8.5%	1
GC 1	ATP5MF	0.001184213	0.442897304	80.9%	59.6%	1
GC 1	CLN6	0.001187443	0.260914354	40.4%	18.1%	1
GC 1	ATIC	0.001226711	0.345689453	59.6%	32.4%	1
GC 1	CORO1A	0.00125213	0.349039941	97.9%	96.3%	1
GC 1	PLEKHA2	0.001256888	0.337034987	57.4%	32.4%	1

GC 1	SDE2	0.001271075	0.290079229	40.4%	18.6%	1
GC 1	TXN	0.00130643	0.261232163	55.3%	28.2%	1
GC 1	PSMA7	0.00135632	0.374509693	83.0%	60.1%	1
GC 1	EIF4H	0.00138277	0.284505936	95.7%	85.6%	1
GC 1	TTC9C	0.001390038	0.263034406	40.4%	18.1%	1
GC 1	RBM8A	0.001433851	0.303577069	59.6%	33.0%	1
GC 1	TRAP1	0.001448587	0.325638414	42.6%	20.7%	1
GC 1	IKBKE	0.001451252	0.309427311	51.1%	25.5%	1
GC 1	CHCHD1	0.001482269	0.487938046	70.2%	47.9%	1
GC 1	MTHFD1	0.001487141	0.253756592	27.7%	10.1%	1
GC 1	DDX56	0.001510173	0.25276607	44.7%	21.3%	1
GC 1	ANKRD13A	0.0015103	0.293731203	48.9%	23.9%	1
GC 1	ARGLU1	0.001553156	0.373814837	95.7%	84.0%	1
GC 1	NSA2	0.001557166	0.337034987	61.7%	36.7%	1
GC 1	PCLAF	0.00157034	0.320787173	48.9%	26.1%	1
GC 1	ATAD1	0.001617014	0.274174963	31.9%	12.8%	1
GC 1	NUBP2	0.001657864	0.284640862	46.8%	22.9%	1
GC 1	FIBP	0.001683803	0.280107919	36.2%	15.4%	1
GC 1	CD82	0.001685956	0.277007348	55.3%	28.7%	1
GC 1	PPP6R1	0.001704855	0.276517635	66.0%	36.2%	1
GC 1	KHDRBS1	0.001749367	0.337928574	57.4%	32.4%	1
GC 1	YWHAQ	0.00177916	0.385550735	70.2%	53.7%	1
GC 1	IMPDH2	0.001780718	0.286562941	38.3%	17.6%	1
GC 1	ANP32A	0.001788613	0.364996817	55.3%	32.4%	1
GC 1	RBM3	0.001796998	0.388297651	97.9%	88.3%	1
GC 1	MAP4K4	0.001817395	0.294743266	38.3%	17.6%	1
GC 1	LSM5	0.001825845	0.298341275	63.8%	36.7%	1
GC 1	SLC25A3	0.001866911	0.327164743	89.4%	72.9%	1
GC 1	MAP3K7CL	0.001898977	0.307624557	34.0%	14.9%	1
GC 1	AMFR	0.001903658	0.258533014	29.8%	11.7%	1
GC 1	ELAVL1	0.001934979	0.354430735	70.2%	48.9%	1
GC 1	VPREB3	0.001979939	0.303577069	53.2%	28.2%	1
GC 1	CCNA2	0.002017676	0.260709353	25.5%	9.0%	1
GC 1	ATXN10	0.002024523	0.397963986	48.9%	27.1%	1
GC 1	RRP1	0.002031787	0.256180735	29.8%	11.7%	1
GC 1	HIST1H3H	0.002038749	0.324002423	91.5%	68.1%	1
GC 1	SMC2	0.00205891	0.254183488	29.8%	11.7%	1
GC 1	EXOSC7	0.002203838	0.25180715	31.9%	13.3%	1
GC 1	MIF	0.002282366	0.320969175	61.7%	34.0%	1
GC 1	TMEM258	0.002364738	0.310700839	53.2%	28.2%	1
GC 1	MCRIP1	0.00238053	0.342468873	51.1%	29.8%	1
GC 1	DOCK2	0.002451044	0.259242827	51.1%	27.1%	1
GC 1	NDUFB6	0.002489847	0.356856428	48.9%	27.7%	1
GC 1	CKAP5	0.002500936	0.25649156	34.0%	14.9%	1
GC 1	CDV3	0.002534342	0.364411426	83.0%	74.5%	1
GC 1	CDK10	0.0026319	0.260709353	25.5%	9.6%	1
GC 1	BET1	0.002701341	0.287281952	40.4%	19.7%	1
GC 1	ZNF318	0.002717639	0.29620127	29.8%	12.2%	1
GC 1	DHFR	0.002728294	0.267695783	25.5%	9.6%	1
GC 1	BCL7A	0.002765816	0.283792966	40.4%	20.2%	1
GC 1	SCAF4	0.002849344	0.25318662	48.9%	25.0%	1
GC 1	PARK7	0.002887621	0.315056618	85.1%	79.3%	1
GC 1	MCM3	0.002930476	0.266280065	61.7%	36.2%	1
GC 1	RAD23A	0.002988708	0.333737397	61.7%	38.3%	1
GC 1	BLMH	0.003045822	0.254183488	34.0%	15.4%	1
GC 1	DCAF16	0.003316422	0.306427001	53.2%	29.3%	1
GC 1	SYVN1	0.003330625	0.35614381	78.7%	56.4%	1
GC 1	CARS2	0.003388064	0.323198632	38.3%	19.1%	1
GC 1	PHGDH	0.003416146	0.286562941	34.0%	15.4%	1
GC 1	CCDC167	0.003424965	0.27064759	48.9%	25.5%	1
GC 1	LASP1	0.003520521	0.390459477	59.6%	39.4%	1
GC 1	LSM8	0.003530259	0.28228229	57.4%	32.4%	1
GC 1	YY1	0.003622916	0.275402963	55.3%	31.9%	1

GC 1	UBA2	0.003692878	0.339850003	48.9%	28.2%	1
GC 1	HADHA	0.003704889	0.304006187	44.7%	24.5%	1
GC 1	COX6C	0.003805841	0.273018494	59.6%	34.6%	1
GC 1	SNRPC	0.004029711	0.32694617	59.6%	37.2%	1
GC 1	ATP5PB	0.004065761	0.386692874	46.8%	27.1%	1
GC 1	UBE2R2	0.004140908	0.296564029	44.7%	23.9%	1
GC 1	SUZ12	0.004239963	0.274556276	59.6%	36.2%	1
GC 1	SRSF2	0.004262217	0.31817596	66.0%	43.1%	1
GC 1	FCHO1	0.004291024	0.26710407	40.4%	20.7%	1
GC 1	MOB1A	0.004361007	0.331896264	80.9%	63.8%	1
GC 1	PPP1CA	0.004437073	0.325005298	55.3%	34.6%	1
GC 1	BRI3BP	0.004490952	0.39665897	63.8%	44.7%	1
GC 1	HELLS	0.004508615	0.255559285	48.9%	26.6%	1
GC 1	EPN1	0.004529741	0.278101854	48.9%	27.7%	1
GC 1	PNOC	0.004529837	0.288957751	40.4%	20.7%	1
GC 1	NUTF2	0.004702691	0.341508464	70.2%	46.8%	1
GC 1	TXNL4A	0.004827489	0.326032493	80.9%	57.4%	1
GC 1	RRAS2	0.005117232	0.293408055	40.4%	21.3%	1
GC 1	RPRD1B	0.005140864	0.278186631	31.9%	14.9%	1
GC 1	DAZAP1	0.005171819	0.298223834	53.2%	31.9%	1
GC 1	BUB3	0.005255818	0.272079545	51.1%	29.3%	1
GC 1	TRA2B	0.0052569	0.362975844	78.7%	64.4%	1
GC 1	DDX54	0.005330433	0.251061764	42.6%	21.8%	1
GC 1	PSMC3	0.005332943	0.330721213	36.2%	18.6%	1
GC 1	AP2S1	0.00569077	0.257035665	42.6%	22.9%	1
GC 1	ACADM	0.00575084	0.257035665	40.4%	20.7%	1
GC 1	GTF2I	0.005786035	0.299560282	68.1%	46.3%	1
GC 1	RGS13	0.005936429	0.323889611	48.9%	27.1%	1
GC 1	SPIB	0.006151886	0.297266041	85.1%	73.4%	1
GC 1	MBD2	0.006380678	0.266393418	57.4%	34.6%	1
GC 1	DDX21	0.006399799	0.306838759	76.6%	53.7%	1
GC 1	CUEDC2	0.006456617	0.255257055	44.7%	23.9%	1
GC 1	RBM14	0.006544937	0.276517635	57.4%	33.5%	1
GC 1	PAG1	0.006573082	0.270357747	51.1%	29.3%	1
GC 1	C12orf75	0.006735085	0.28228229	48.9%	28.2%	1
GC 1	GTF3C6	0.006956653	0.252516534	83.0%	64.9%	1
GC 1	PSMC5	0.007182824	0.275402963	55.3%	34.0%	1
GC 1	GLRX5	0.007239225	0.316184112	59.6%	39.4%	1
GC 1	POLD2	0.007332923	0.346450414	55.3%	33.5%	1
GC 1	CDC123	0.00735402	0.311944006	55.3%	34.6%	1
GC 1	SRSF7	0.007527064	0.406159348	66.0%	50.0%	1
GC 1	HSBP1	0.007646242	0.318419309	44.7%	26.6%	1
GC 1	HNRNPU	0.007648617	0.302269569	53.2%	33.0%	1
GC 1	RUBCNL	0.007785864	0.311944006	57.4%	36.2%	1
GC 1	ACO2	0.007799142	0.354802392	51.1%	33.5%	1
GC 1	POLA2	0.007817053	0.281181753	38.3%	20.2%	1
GC 1	CWC15	0.00811004	0.276935223	42.6%	24.5%	1
GC 1	CALM3	0.008129517	0.346228341	72.3%	59.0%	1
GC 1	DENR	0.008132828	0.250543462	40.4%	21.8%	1
GC 1	TOP2B	0.008433455	0.324556753	66.0%	50.0%	1
GC 1	POLR2G	0.008439578	0.302360726	76.6%	66.0%	1
GC 1	BRWD1	0.008485868	0.306427001	51.1%	32.4%	1
GC 1	C4orf3	0.008627922	0.293445719	93.6%	81.9%	1
GC 1	MEPCE	0.008961904	0.261086134	44.7%	25.0%	1
GC 1	NDUFC1	0.008975833	0.288957751	40.4%	22.9%	1
GC 1	UHRF1	0.009143898	0.260914354	34.0%	17.6%	1
GC 1	SF3B5	0.009158328	0.286304185	42.6%	25.0%	1
GC 1	MRFAP1L1	0.009236572	0.297614638	70.2%	51.1%	1
GC 1	ZWINT	0.009353256	0.252307999	31.9%	15.4%	1
GC 1	NDUFB7	0.009586021	0.304006187	68.1%	46.3%	1
GC 1	MKI67	0.00965879	0.287925581	42.6%	24.5%	1
GC 1	RCC2	0.009766878	0.27064759	78.7%	60.1%	1
GC 2	RGS13	3.88E-18	1.164300452	83.7%	19.8%	5.86E-14

GC 2	HIST1H3H	4.69E-15	1.217591435	100.0%	66.7%	7.08E-11
GC 2	HIST1H4E	5.34E-15	0.946153111	90.7%	33.9%	8.06E-11
GC 2	HIST1H2AG	3.69E-14	1.162220813	95.3%	57.8%	5.57E-10
GC 2	UCP2	1.26E-13	0.902599387	95.3%	48.4%	1.90E-09
GC 2	HIST1H2BH	1.64E-13	1.11104477	79.1%	28.1%	2.48E-09
GC 2	BCL6	1.98E-13	0.906914475	86.0%	31.2%	2.99E-09
GC 2	CLU	7.59E-13	1.809062722	97.7%	86.5%	1.15E-08
GC 2	HMGB1	3.15E-12	0.937644253	95.3%	55.7%	4.76E-08
GC 2	HIST1H2BO	7.36E-12	0.959626399	97.7%	58.3%	1.11E-07
GC 2	HIST1H4C	8.39E-12	0.997040361	100.0%	83.3%	1.27E-07
GC 2	CR2	8.80E-12	1.290380855	95.3%	62.5%	1.33E-07
GC 2	MYBL2	1.53E-11	0.884775024	76.7%	29.2%	2.30E-07
GC 2	HIST1H2BG	1.93E-11	0.897499999	86.0%	42.7%	2.91E-07
GC 2	KLHL6	2.38E-11	0.775092755	95.3%	66.1%	3.59E-07
GC 2	POLD4	2.95E-11	0.86401089	90.7%	49.5%	4.46E-07
GC 2	HIST1H1B	5.70E-11	0.950316434	100.0%	84.9%	8.61E-07
GC 2	LTF	7.18E-11	0.794541416	72.1%	21.9%	1.08E-06
GC 2	HIST1H3G	1.50E-10	0.924016536	100.0%	74.0%	2.27E-06
GC 2	E2F5	1.30E-09	0.514028999	55.8%	14.1%	1.96E-05
GC 2	CD81	1.93E-09	0.62976928	95.3%	71.9%	2.91E-05
GC 2	MCM5	2.07E-09	0.588090538	90.7%	36.5%	3.13E-05
GC 2	CRIP1	3.57E-09	0.694416728	83.7%	35.4%	5.39E-05
GC 2	MARCKSL1	3.99E-09	0.864752064	90.7%	65.6%	6.02E-05
GC 2	EIF2S3	5.97E-09	0.655265131	69.8%	27.6%	9.01E-05
GC 2	HIST2H2AB	6.75E-09	0.850012228	97.7%	60.9%	0.000101948
GC 2	OAZ1	7.24E-09	0.606487958	100.0%	95.8%	0.0001092
GC 2	XRCC6	7.32E-09	0.584962501	69.8%	24.0%	0.000110468
GC 2	RUVBL2	1.82E-08	0.555587899	60.5%	19.3%	0.000274851
GC 2	HIST1H2BM	2.02E-08	0.768551422	62.8%	24.0%	0.000304517
GC 2	HIST1H2AI	2.10E-08	0.836769651	88.4%	55.2%	0.000316431
GC 2	CD22	2.49E-08	0.663094765	86.0%	42.2%	0.000375901
GC 2	ANP32B	3.05E-08	0.787016616	72.1%	33.9%	0.000460525
GC 2	CCDC88A	3.09E-08	0.606156723	79.1%	32.3%	0.000466752
GC 2	HIST1H3I	5.00E-08	0.677291437	93.0%	60.9%	0.000755054
GC 2	CD79B	8.16E-08	0.677892477	90.7%	59.4%	0.001231639
GC 2	MEF2B	1.04E-07	0.719586112	95.3%	66.1%	0.00157629
GC 2	RFTN1	1.24E-07	0.667162109	69.8%	30.2%	0.001868604
GC 2	MAST2	1.24E-07	0.271908356	23.3%	2.1%	0.001870843
GC 2	RAP1A	1.29E-07	0.618129365	76.7%	33.3%	0.001948375
GC 2	NONO	1.36E-07	0.523572451	100.0%	87.0%	0.002048167
GC 2	RBM17	1.76E-07	0.587674436	83.7%	40.1%	0.002659015
GC 2	SYNE2	1.96E-07	0.640461359	86.0%	45.3%	0.002955097
GC 2	GALNT14	1.99E-07	0.25180715	20.9%	1.6%	0.002997519
GC 2	TCL1A	2.39E-07	0.612729377	95.3%	57.8%	0.003604337
GC 2	CENPE	2.39E-07	0.402934556	32.6%	5.7%	0.003607783
GC 2	PCLAF	2.40E-07	0.47948492	65.1%	22.9%	0.003620558
GC 2	MYO1E	2.56E-07	0.375189566	39.5%	8.9%	0.003870311
GC 2	HIST1H2AE	2.72E-07	0.692298443	95.3%	65.1%	0.004098066
GC 2	DAZAP1	2.72E-07	0.501999492	72.1%	28.1%	0.004101398
GC 2	ELL3	3.01E-07	0.568021594	62.8%	24.0%	0.004536588
GC 2	HIST1H4I	3.26E-07	0.664883133	95.3%	68.8%	0.004925907
GC 2	HIST1H2BE	3.33E-07	0.382075952	34.9%	6.8%	0.005033213
GC 2	HSPE1	3.46E-07	0.600069393	88.4%	55.7%	0.005217549
GC 2	SYVN1	3.52E-07	0.608500663	86.0%	55.2%	0.005305296
GC 2	TPX2	3.60E-07	0.588234815	74.4%	33.3%	0.005435043
GC 2	SRGN	3.89E-07	0.646585811	62.8%	26.0%	0.005865325
GC 2	CTSH	5.89E-07	0.602590108	74.4%	40.1%	0.008896582
GC 2	SNCG	6.72E-07	0.326251843	30.2%	5.2%	0.010140732
GC 2	SWAP70	6.78E-07	0.503099703	76.7%	34.9%	0.010231019
GC 2	CCNI	6.91E-07	0.573735245	95.3%	70.8%	0.010433952
GC 2	LAP3	7.14E-07	0.536741038	53.5%	18.8%	0.010779005
GC 2	HIST1H2BL	7.61E-07	0.436231722	53.5%	17.7%	0.011479479
GC 2	MIF	9.52E-07	0.693034174	67.4%	33.3%	0.014375545

GC 2	DBI	9.72E-07	0.511807496	88.4%	54.2%	0.014672084
GC 2	IRF8	1.08E-06	0.703957823	74.4%	39.1%	0.016293142
GC 2	ATP5MG	1.16E-06	0.559863071	90.7%	70.3%	0.017532237
GC 2	SCARA3	1.20E-06	0.388995365	34.9%	7.8%	0.018165415
GC 2	HIST1H3D	1.26E-06	0.627398956	100.0%	81.8%	0.01903787
GC 2	PLCG2	1.36E-06	0.605796455	65.1%	31.2%	0.02056479
GC 2	ISG20	1.65E-06	0.561024194	83.7%	48.4%	0.024879438
GC 2	FTL	1.96E-06	0.481497297	97.7%	87.5%	0.02955315
GC 2	CD27	2.20E-06	0.552282136	65.1%	28.1%	0.033263697
GC 2	NUCKS1	2.63E-06	0.58109595	88.4%	66.7%	0.03972458
GC 2	KHSRP	2.80E-06	0.55275022	67.4%	30.7%	0.04220532
GC 2	SERPINA9	2.81E-06	0.649684099	58.1%	24.5%	0.04237271
GC 2	UBB	2.84E-06	0.564604234	76.7%	44.3%	0.042910583
GC 2	MYBL1	3.00E-06	0.511807496	51.2%	18.2%	0.045255973
GC 2	HIST1H2BD	3.05E-06	0.533093261	93.0%	66.1%	0.046035047
GC 2	TP53	3.14E-06	0.393163	44.2%	13.5%	0.04738298
GC 2	PAQR4	3.42E-06	0.269729058	25.6%	4.2%	0.051673849
GC 2	LMO2	3.77E-06	0.688751906	48.8%	18.2%	0.056955675
GC 2	ARHGAP11A	3.81E-06	0.377338032	44.2%	13.5%	0.05747905
GC 2	PAG1	3.98E-06	0.54539062	60.5%	27.6%	0.06007253
GC 2	SEL1L3	5.08E-06	0.504294747	97.7%	77.6%	0.076714874
GC 2	ACTR3	6.23E-06	0.434245906	100.0%	93.2%	0.094091635
GC 2	HIST1H2BI	6.28E-06	0.61437723	93.0%	64.6%	0.094804306
GC 2	CSTA	6.54E-06	0.364701207	39.5%	11.5%	0.09863635
GC 2	HNRNPC	6.72E-06	0.500720448	86.0%	60.4%	0.101468078
GC 2	TFDP1	6.73E-06	0.490479742	79.1%	43.8%	0.101536571
GC 2	NDC80	6.85E-06	0.414878481	34.9%	8.9%	0.103442692
GC 2	DNMT1	7.10E-06	0.440733114	88.4%	48.4%	0.107210274
GC 2	HIST1H2AD	7.33E-06	0.526195687	97.7%	75.5%	0.110686718
GC 2	CSK	7.46E-06	0.56079619	76.7%	49.5%	0.112609642
GC 2	PLEKHF2	7.47E-06	0.438475416	60.5%	25.0%	0.112815492
GC 2	HIST1H2BB	7.61E-06	0.63467801	86.0%	51.6%	0.114878875
GC 2	PEPD	7.79E-06	0.315000977	25.6%	4.7%	0.117632464
GC 2	THOC7	7.83E-06	0.494746949	55.8%	23.4%	0.118108447
GC 2	MCM3	8.31E-06	0.528411255	69.8%	34.9%	0.125493999
GC 2	C12orf75	8.42E-06	0.552040174	60.5%	26.0%	0.127079428
GC 2	HSP90AB1	8.63E-06	0.58033798	90.7%	68.2%	0.130189006
GC 2	C4orf46	9.10E-06	0.300716751	27.9%	5.7%	0.137290646
GC 2	TOP2A	9.46E-06	0.354332867	51.2%	17.2%	0.14276681
GC 2	HIST1H4H	9.99E-06	0.621871766	95.3%	71.4%	0.150752241
GC 2	HMCES	1.00E-05	0.415189306	41.9%	13.5%	0.150901001
GC 2	CDCA7	1.03E-05	0.508640217	88.4%	51.0%	0.155796807
GC 2	RAPGEF5	1.04E-05	0.361514629	34.9%	9.4%	0.156479778
GC 2	FCRL2	1.05E-05	0.388179592	51.2%	19.3%	0.159062721
GC 2	TMED8	1.12E-05	0.496567385	69.8%	34.4%	0.168666768
GC 2	PTEN	1.12E-05	0.310700839	39.5%	11.5%	0.169058742
GC 2	NUGGC	1.18E-05	0.49054131	46.5%	16.7%	0.177487959
GC 2	AP2M1	1.23E-05	0.466401201	65.1%	31.2%	0.18547761
GC 2	NANS	1.27E-05	0.440110681	60.5%	26.0%	0.1916104
GC 2	STAP1	1.30E-05	0.377338032	41.9%	13.5%	0.196783995
GC 2	MAP4K4	1.32E-05	0.370201851	46.5%	16.1%	0.198950243
GC 2	LPP	1.35E-05	0.417616043	48.8%	18.2%	0.204221986
GC 2	HIST1H1E	1.37E-05	0.500803723	100.0%	96.4%	0.20705651
GC 2	SIT1	1.47E-05	0.421732152	41.9%	14.1%	0.222336172
GC 2	MKNK2	1.56E-05	0.511271158	83.7%	53.1%	0.234883407
GC 2	FAM49B	1.67E-05	0.452105801	48.8%	19.3%	0.252059198
GC 2	TMEM243	1.70E-05	0.383632362	55.8%	21.9%	0.256831025
GC 2	VGLL4	1.84E-05	0.323191784	30.2%	7.3%	0.278050779
GC 2	CPNE5	1.85E-05	0.463552328	62.8%	27.6%	0.278636983
GC 2	DCK	1.85E-05	0.47455984	69.8%	35.9%	0.279622634
GC 2	MCM2	1.89E-05	0.377338032	39.5%	12.5%	0.285098526
GC 2	SYK	1.91E-05	0.44396737	67.4%	33.3%	0.288099334
GC 2	VCAM1	2.01E-05	0.269729058	23.3%	4.2%	0.303756056

GC 2	MOB3A	2.14E-05	0.454522087	69.8%	35.4%	0.32259486
GC 2	SPTAN1	2.22E-05	0.337667887	34.9%	9.9%	0.334454448
GC 2	CCT7	2.25E-05	0.380255516	55.8%	22.9%	0.339072219
GC 2	NCOA3	2.28E-05	0.520689011	62.8%	31.2%	0.343949801
GC 2	DTX1	2.35E-05	0.52734234	72.1%	38.5%	0.355403228
GC 2	CR1	2.55E-05	0.333981663	32.6%	8.9%	0.384842962
GC 2	GNG2	2.55E-05	0.341242234	34.9%	9.9%	0.385288673
GC 2	BPTF	2.98E-05	0.438805665	69.8%	34.9%	0.449569324
GC 2	MAZ	3.21E-05	0.503345917	55.8%	24.5%	0.484052745
GC 2	CD40	3.45E-05	0.484925978	62.8%	32.8%	0.52063599
GC 2	H2AFV	3.59E-05	0.568734564	62.8%	34.9%	0.542281189
GC 2	HIST1H3A	3.65E-05	0.410676963	58.1%	25.0%	0.551214405
GC 2	NME1	3.84E-05	0.45692158	60.5%	28.6%	0.579943986
GC 2	FGD6	3.93E-05	0.330178214	27.9%	6.8%	0.593485982
GC 2	APOC1	3.94E-05	0.312107661	27.9%	6.8%	0.59539468
GC 2	CCT5	4.01E-05	0.46110639	55.8%	25.5%	0.605204493
GC 2	HIST1H4B	4.02E-05	0.483132697	88.4%	65.1%	0.606720887
GC 2	NIN	4.26E-05	0.396389304	62.8%	29.7%	0.642416943
GC 2	DESI2	4.40E-05	0.324991497	37.2%	11.5%	0.664615369
GC 2	HSPA8	4.69E-05	0.514459436	93.0%	71.4%	0.708594494
GC 2	FAM3C	4.84E-05	0.504194312	55.8%	26.6%	0.730952913
GC 2	COX7C	4.98E-05	0.401141057	95.3%	84.4%	0.751313167
GC 2	SERPINA3	5.14E-05	0.262533557	23.3%	4.7%	0.776421813
GC 2	YEATS2	5.38E-05	0.299873809	34.9%	10.4%	0.812209277
GC 2	IKZF3	5.53E-05	0.302433223	32.6%	9.4%	0.834021416
GC 2	SMAP2	5.84E-05	0.462703933	74.4%	43.8%	0.881495524
GC 2	PSMC3	5.85E-05	0.29842251	46.5%	16.7%	0.882518576
GC 2	STX11	6.04E-05	0.311994156	39.5%	13.0%	0.911379364
GC 2	AFF2	6.77E-05	0.396389304	60.5%	27.1%	1
GC 2	ATP2A3	6.94E-05	0.418564873	74.4%	42.2%	1
GC 2	MCM4	7.66E-05	0.297404017	37.2%	12.0%	1
GC 2	GCHFR	7.98E-05	0.480625841	48.8%	21.9%	1
GC 2	IQCB1	8.43E-05	0.320241607	32.6%	9.9%	1
GC 2	STK17B	9.48E-05	0.404397541	74.4%	39.6%	1
GC 2	POU2AF1	9.79E-05	0.438662548	95.3%	82.3%	1
GC 2	CCDC69	0.000102844	0.430315998	86.0%	58.9%	1
GC 2	GDI2	0.000106382	0.393163	69.8%	36.5%	1
GC 2	ARHGDIB	0.000110611	0.387139212	100.0%	95.8%	1
GC 2	TKT	0.000113845	0.4536414	69.8%	40.6%	1
GC 2	ARPC4	0.000115089	0.381090167	95.3%	85.9%	1
GC 2	SAP18	0.000117868	0.345293779	48.8%	20.3%	1
GC 2	FAM107B	0.000120928	0.384579153	46.5%	19.3%	1
GC 2	RNF144B	0.000125812	0.48433616	44.2%	17.7%	1
GC 2	LRRK1	0.000134859	0.379296886	39.5%	14.6%	1
GC 2	SF3B6	0.000136566	0.324707697	53.5%	22.9%	1
GC 2	METAP2	0.000137111	0.368915453	90.7%	53.1%	1
GC 2	RNPS1	0.000139527	0.332149259	39.5%	14.6%	1
GC 2	ODC1	0.000144604	0.495732733	76.7%	51.6%	1
GC 2	TADA3	0.000152872	0.408675999	37.2%	14.1%	1
GC 2	MZB1	0.00016095	0.4367996	53.5%	25.0%	1
GC 2	SUSD3	0.000168384	0.333271251	48.8%	20.3%	1
GC 2	RASGRP3	0.000168517	0.368151112	53.5%	24.0%	1
GC 2	HNRNPAB	0.00017192	0.366502631	65.1%	33.9%	1
GC 2	PAPSS1	0.000175104	0.304548612	44.2%	17.2%	1
GC 2	LSM2	0.000177362	0.3118976	48.8%	20.3%	1
GC 2	CAMK1	0.000181238	0.365148623	27.9%	8.3%	1
GC 2	CYTIP	0.000195465	0.345293779	48.8%	21.4%	1
GC 2	PHB2	0.000203957	0.402623329	69.8%	41.1%	1
GC 2	DCAF12	0.00020647	0.403116474	53.5%	24.0%	1
GC 2	CDC27	0.000214092	0.258693536	27.9%	7.8%	1
GC 2	PSMA2	0.000218339	0.332727146	44.2%	17.7%	1
GC 2	UBE2J1	0.000218525	0.369264732	60.5%	30.7%	1
GC 2	PBK	0.000225251	0.295579552	30.2%	9.4%	1

GC 2	SMIM27	0.000238698	0.25180715	27.9%	7.8%	1
GC 2	C19orf53	0.000238835	0.313025892	51.2%	21.9%	1
GC 2	ALYREF	0.000245638	0.396607558	51.2%	22.4%	1
GC 2	RHOH	0.000258333	0.339938061	55.8%	26.6%	1
GC 2	SGSM3	0.000260422	0.270488571	30.2%	9.4%	1
GC 2	ACTB	0.000269563	0.268670588	100.0%	100.0%	1
GC 2	DEK	0.000276927	0.474881858	97.7%	81.8%	1
GC 2	MYH9	0.00027765	0.345203687	95.3%	73.4%	1
GC 2	HNRNPA3	0.000283025	0.40814918	100.0%	99.5%	1
GC 2	RBBP7	0.000295391	0.428018415	39.5%	15.6%	1
GC 2	C19orf48	0.000297592	0.373037895	41.9%	17.7%	1
GC 2	CDT1	0.000305097	0.423631685	48.8%	22.4%	1
GC 2	ACY3	0.000311285	0.26561295	27.9%	8.3%	1
GC 2	CUL3	0.000342236	0.295019217	37.2%	13.5%	1
GC 2	RABGAP1L	0.00034894	0.358996397	62.8%	33.9%	1
GC 2	TROAP	0.000370872	0.280004042	41.9%	16.7%	1
GC 2	LNPEP	0.000379795	0.278279362	48.8%	21.4%	1
GC 2	RAC2	0.000384994	0.417616043	79.1%	56.2%	1
GC 2	CLDN16	0.000391703	0.298100803	25.6%	7.3%	1
GC 2	UGGT1	0.000394384	0.375189566	27.9%	8.9%	1
GC 2	YWHAE	0.000404925	0.299873809	30.2%	9.9%	1
GC 2	BCAS4	0.000407473	0.362838463	81.4%	52.1%	1
GC 2	PCNA	0.000422263	0.323920128	44.2%	18.8%	1
GC 2	HSP90AA1	0.000430061	0.361627426	95.3%	85.4%	1
GC 2	GLUL	0.000436337	0.43171624	65.1%	35.9%	1
GC 2	SF3B5	0.000436744	0.263167013	53.5%	22.9%	1
GC 2	GPNMB	0.000443985	0.258693536	25.6%	7.3%	1
GC 2	SERBP1	0.000467715	0.384464894	79.1%	53.6%	1
GC 2	TK1	0.000468855	0.297404017	32.6%	11.5%	1
GC 2	PABPC1L	0.000479797	0.361514629	30.2%	10.4%	1
GC 2	VAMP1	0.000491298	0.348175545	41.9%	18.2%	1
GC 2	FKBP1A	0.000495701	0.357601814	39.5%	16.1%	1
GC 2	MBD2	0.000504458	0.425091164	60.5%	34.4%	1
GC 2	PSMA5	0.000511069	0.322196478	53.5%	25.5%	1
GC 2	OGG1	0.000520377	0.287980763	58.1%	28.6%	1
GC 2	NUSAP1	0.000526816	0.33472811	53.5%	25.0%	1
GC 2	RAB8B	0.000541787	0.250120774	30.2%	9.9%	1
GC 2	ENTPD4	0.000557644	0.386108242	48.8%	23.4%	1
GC 2	EIF3H	0.000568001	0.319162418	58.1%	28.1%	1
GC 2	LCOR	0.000568822	0.313207695	41.9%	18.2%	1
GC 2	USP7	0.000617749	0.328110518	74.4%	44.8%	1
GC 2	CSE1L	0.000622809	0.366805941	41.9%	19.3%	1
GC 2	CORO1A	0.000623108	0.28862805	100.0%	95.8%	1
GC 2	LYN	0.000626919	0.357417338	44.2%	20.3%	1
GC 2	SUMO1	0.000645937	0.282356214	53.5%	24.5%	1
GC 2	PAX5	0.000675655	0.353384855	76.7%	52.1%	1
GC 2	TOP1	0.000679079	0.322196478	76.7%	47.9%	1
GC 2	ATOX1	0.000686252	0.363168259	44.2%	20.3%	1
GC 2	NAA38	0.000699059	0.275021864	58.1%	29.2%	1
GC 2	MZT1	0.000733875	0.351342824	41.9%	18.2%	1
GC 2	HIST1H2AB	0.000736845	0.474793396	67.4%	40.1%	1
GC 2	NCAPD2	0.00074005	0.44396737	58.1%	31.2%	1
GC 2	MTPN	0.000741932	0.267926816	60.5%	29.7%	1
GC 2	PPP1CA	0.000748989	0.328622747	60.5%	33.9%	1
GC 2	FAM76B	0.000773412	0.288535184	37.2%	15.1%	1
GC 2	PXK	0.000829101	0.326574919	41.9%	18.8%	1
GC 2	ELAVL1	0.000834142	0.337963794	76.7%	47.9%	1
GC 2	CDV3	0.000858413	0.310700839	95.3%	71.9%	1
GC 2	NCOR2	0.000880505	0.390871188	48.8%	25.5%	1
GC 2	ATP5MC3	0.000885647	0.271791525	41.9%	17.7%	1
GC 2	RALY	0.000887863	0.441097477	60.5%	33.9%	1
GC 2	SF3B1	0.000911775	0.329683943	67.4%	41.1%	1
GC 2	MKI67	0.000924457	0.319760179	48.8%	23.4%	1

GC 2	PPP6R1	0.000936893	0.323622066	65.1%	37.0%	1
GC 2	NFYC	0.000956869	0.29232231	41.9%	18.2%	1
GC 2	SCARB1	0.000974002	0.275211755	30.2%	10.9%	1
GC 2	SEPTIN9	0.00101752	0.276054697	41.9%	17.7%	1
GC 2	RAB13	0.001028051	0.279789563	32.6%	12.5%	1
GC 2	RALA	0.001036758	0.25823342	37.2%	15.1%	1
GC 2	UBA2	0.001046705	0.329232122	53.5%	27.6%	1
GC 2	DDT	0.001094948	0.378010774	55.8%	29.7%	1
GC 2	RHOA	0.001112657	0.294242417	97.7%	93.8%	1
GC 2	SEMA4A	0.001126633	0.268176223	46.5%	21.9%	1
GC 2	SIAH2	0.001128463	0.344916555	34.9%	14.6%	1
GC 2	LCP1	0.00114004	0.37064338	62.8%	39.6%	1
GC 2	PPM1A	0.001154156	0.25180715	37.2%	15.1%	1
GC 2	GPR52	0.001160176	0.267404005	39.5%	16.7%	1
GC 2	AZIN1	0.001172703	0.391143832	62.8%	37.5%	1
GC 2	CCL18	0.001190937	0.297404017	30.2%	10.9%	1
GC 2	SOCS1	0.001206917	0.255127509	32.6%	12.5%	1
GC 2	ARPC1B	0.001283497	0.256443561	97.7%	94.8%	1
GC 2	DDB1	0.001318259	0.345466258	48.8%	25.0%	1
GC 2	SNAP23	0.001346654	0.30807537	32.6%	12.5%	1
GC 2	TRABD	0.00134771	0.300053595	60.5%	32.8%	1
GC 2	POLA2	0.001403519	0.250328221	44.2%	19.3%	1
GC 2	EIF4G2	0.001467078	0.428489518	74.4%	59.4%	1
GC 2	PTK2	0.001467656	0.377043334	65.1%	40.6%	1
GC 2	SERP1	0.001467688	0.28743106	67.4%	37.5%	1
GC 2	LMNB1	0.001503198	0.300716751	39.5%	17.7%	1
GC 2	EHD1	0.001521114	0.353713728	65.1%	41.7%	1
GC 2	RRAS2	0.001531638	0.290202529	44.2%	20.8%	1
GC 2	HMGN3	0.001543703	0.322998198	51.2%	26.6%	1
GC 2	KHDRBS1	0.001595521	0.287684187	58.1%	32.8%	1
GC 2	CCNB2	0.001604922	0.277685782	32.6%	13.0%	1
GC 2	ATF5	0.001751398	0.293147946	30.2%	12.0%	1
GC 2	GOT2	0.001759517	0.269256121	34.9%	14.6%	1
GC 2	EEF1G	0.001769602	0.320921731	97.7%	89.1%	1
GC 2	NDUFB10	0.001786611	0.286158655	44.2%	20.3%	1
GC 2	NEIL1	0.001789375	0.284228628	41.9%	19.3%	1
GC 2	OXA1L	0.001792007	0.269954497	44.2%	21.4%	1
GC 2	HNRNPR	0.001808877	0.333531548	76.7%	47.4%	1
GC 2	NFATC1	0.001818081	0.302225057	41.9%	19.3%	1
GC 2	PSMB8	0.001960232	0.275021864	53.5%	28.1%	1
GC 2	MCM7	0.001985091	0.299112865	88.4%	63.5%	1
GC 2	DUT	0.002005041	0.38717493	72.1%	49.5%	1
GC 2	TBRG4	0.002007207	0.311994156	32.6%	13.0%	1
GC 2	TBL1XR1	0.002038943	0.351342824	46.5%	25.0%	1
GC 2	FANCA	0.002123017	0.331714017	46.5%	24.0%	1
GC 2	SMG7	0.002135107	0.267632118	20.9%	6.2%	1
GC 2	NCF4	0.002172976	0.277342243	25.6%	8.9%	1
GC 2	SFPQ	0.002412184	0.303337451	55.8%	31.2%	1
GC 2	TFIP11	0.002430456	0.259981082	32.6%	13.5%	1
GC 2	GNAI2	0.002485536	0.325516837	65.1%	41.1%	1
GC 2	NUTF2	0.002518659	0.307220271	72.1%	46.9%	1
GC 2	ATP5MF	0.002557884	0.300716751	83.7%	59.4%	1
GC 2	LAMTOR1	0.002633808	0.288148997	44.2%	21.9%	1
GC 2	DEF8	0.002639372	0.382912903	34.9%	15.6%	1
GC 2	CCND3	0.002651981	0.31542325	41.9%	20.3%	1
GC 2	CTSB	0.002700932	0.348442933	46.5%	24.5%	1
GC 2	UBA1	0.002761133	0.275653892	34.9%	15.1%	1
GC 2	MOB1A	0.002780733	0.290428444	86.0%	63.0%	1
GC 2	AURKB	0.002795447	0.313207695	37.2%	17.7%	1
GC 2	SPARC	0.002945913	0.37064338	58.1%	35.4%	1
GC 2	TERF2	0.002963736	0.306631175	27.9%	10.9%	1
GC 2	ADA	0.003084241	0.268176223	41.9%	20.3%	1
GC 2	SNRNP40	0.003154509	0.286247796	41.9%	20.8%	1

GC 2	MCM6	0.003294723	0.292248076	62.8%	35.9%	1
GC 2	WDR76	0.003399157	0.281969121	27.9%	10.9%	1
GC 2	TRAPPC5	0.003480015	0.300716751	60.5%	36.5%	1
GC 2	RBX1	0.003524471	0.369376747	65.1%	46.4%	1
GC 2	PGLS	0.003583846	0.322196478	46.5%	26.0%	1
GC 2	HNRNPF	0.003620479	0.305993837	67.4%	42.7%	1
GC 2	LSM8	0.003634242	0.301362975	55.8%	33.3%	1
GC 2	CD82	0.003680461	0.458258028	51.2%	30.2%	1
GC 2	MCRS1	0.003738645	0.264688442	32.6%	14.1%	1
GC 2	PSME2	0.003785459	0.29620127	55.8%	32.3%	1
GC 2	USF2	0.003817758	0.368335895	76.7%	68.2%	1
GC 2	TACC1	0.003903365	0.271791525	37.2%	17.2%	1
GC 2	MAPKAPK2	0.003925	0.261145015	37.2%	17.2%	1
GC 2	HDAC1	0.004070701	0.288758287	67.4%	39.1%	1
GC 2	CPSF6	0.004073909	0.30745518	55.8%	32.3%	1
GC 2	PPP1R15B	0.004098059	0.293239869	53.5%	29.7%	1
GC 2	PRKACA	0.004110423	0.286158655	44.2%	22.4%	1
GC 2	AIP	0.004121376	0.253243383	46.5%	24.0%	1
GC 2	VASP	0.004152475	0.296625654	67.4%	40.6%	1
GC 2	NSMF	0.004152731	0.264688442	32.6%	14.6%	1
GC 2	CLIC4	0.004159728	0.262886596	32.6%	14.1%	1
GC 2	FCRLA	0.004217816	0.277996674	34.9%	15.6%	1
GC 2	BABAM1	0.004247733	0.282180799	39.5%	19.3%	1
GC 2	TRA2B	0.004306637	0.319162418	83.7%	63.5%	1
GC 2	UBQLN1	0.004326778	0.266553736	30.2%	13.0%	1
GC 2	UBE2E1	0.004437671	0.270590626	44.2%	22.9%	1
GC 2	SNRPG	0.004447755	0.302607157	55.8%	33.3%	1
GC 2	DCUN1D1	0.00447135	0.333784453	44.2%	24.0%	1
GC 2	TCP1	0.004480981	0.311572295	72.1%	47.9%	1
GC 2	ACADM	0.004527152	0.309522648	41.9%	20.8%	1
GC 2	CALR	0.004606219	0.379724625	65.1%	41.7%	1
GC 2	CXCR5	0.004674977	0.262202322	41.9%	20.8%	1
GC 2	MATN2	0.004721462	0.293147946	27.9%	12.0%	1
GC 2	ABI1	0.004799948	0.284228628	37.2%	17.7%	1
GC 2	POLDIP3	0.004916379	0.267222203	48.8%	25.0%	1
GC 2	PCBP2	0.004954312	0.337922308	79.1%	62.5%	1
GC 2	HPCAL1	0.005027791	0.303646082	44.2%	24.5%	1
GC 2	KPNA2	0.005098918	0.276054697	58.1%	35.4%	1
GC 2	RBM38	0.005196504	0.304548612	55.8%	31.2%	1
GC 2	TAP2	0.005222958	0.26561295	39.5%	20.3%	1
GC 2	NHP2	0.005227343	0.301302141	67.4%	39.6%	1
GC 2	ZNF655	0.005333589	0.273156471	30.2%	13.0%	1
GC 2	STRAP	0.005656214	0.309603316	51.2%	29.7%	1
GC 2	EPS15	0.005659768	0.275211755	25.6%	10.4%	1
GC 2	CAPG	0.005747197	0.310700839	46.5%	25.0%	1
GC 2	RNH1	0.005862621	0.320448816	37.2%	18.8%	1
GC 2	PRDX6	0.005938167	0.315937488	67.4%	44.3%	1
GC 2	CCNC	0.006002339	0.256545069	34.9%	16.7%	1
GC 2	FCRL3	0.006045063	0.312503082	69.8%	43.8%	1
GC 2	BACH2	0.006114719	0.397146514	27.9%	12.5%	1
GC 2	MMADHC	0.006144303	0.250328221	44.2%	23.4%	1
GC 2	WDR1	0.006158141	0.314568657	62.8%	42.7%	1
GC 2	UBL5	0.006302895	0.26145732	62.8%	40.6%	1
GC 2	EDEM1	0.006823203	0.254913061	34.9%	17.2%	1
GC 2	TIMM13	0.006911382	0.25180715	46.5%	25.0%	1
GC 2	RFX5	0.006919971	0.289484354	53.5%	31.8%	1
GC 2	LSM7	0.007034054	0.289484354	51.2%	29.7%	1
GC 2	EZR	0.007240155	0.308444866	46.5%	26.6%	1
GC 2	GADD45GIP1	0.007299031	0.322817126	67.4%	50.5%	1
GC 2	RNF4	0.007448719	0.289942279	44.2%	23.4%	1
GC 2	PIK3CG	0.007526119	0.275653892	32.6%	15.1%	1
GC 2	OGT	0.007582588	0.321250759	27.9%	12.5%	1
GC 2	ZFP91	0.007873697	0.25180715	46.5%	25.5%	1

GC 2	FN1	0.007941346	0.284228628	34.9%	17.7%	1
GC 2	HINT1	0.008273844	0.274890764	65.1%	43.2%	1
GC 2	PRRC2A	0.008573164	0.272653935	58.1%	34.4%	1
GC 2	CDC42EP4	0.008960311	0.293147946	25.6%	10.9%	1
GC 2	SUZ12	0.008983917	0.256624167	60.5%	36.5%	1
GC 2	PPDPF	0.009226639	0.263115641	81.4%	69.8%	1
GC 2	ILF3	0.009334503	0.26031865	90.7%	88.0%	1
GC 2	RRP7A	0.009592918	0.346324749	55.8%	34.4%	1
GC 2	HIST1H2BJ	0.009668398	0.291636578	44.2%	25.0%	1
GC 2	SCAF11	0.009771995	0.267404005	32.6%	15.6%	1
GC 2	STX7	0.009965928	0.254408946	58.1%	35.9%	1
T cell zone	CCL21	1.16E-12	1.411085509	54.8%	9.8%	1.75E-08
T cell zone	C7	1.19E-11	0.540794917	35.7%	3.1%	1.80E-07
T cell zone	CD226	1.33E-10	0.293249019	23.8%	0.5%	2.00E-06
T cell zone	IGFBP4	8.59E-10	0.41426442	33.3%	3.6%	1.30E-05
T cell zone	CCL19	9.48E-10	1.461185467	54.8%	16.1%	1.43E-05
T cell zone	IL7R	1.01E-09	0.672510289	50.0%	11.4%	1.52E-05
T cell zone	TRAJ38	1.59E-09	0.430437234	40.5%	6.2%	2.39E-05
T cell zone	DGKA	7.54E-09	0.561984175	47.6%	10.9%	0.000113859
T cell zone	TAGLN	1.63E-08	0.573356938	40.5%	8.3%	0.000245918
T cell zone	CD69	5.51E-08	0.360604287	33.3%	5.2%	0.000831193
T cell zone	TXNIP	6.12E-08	0.640712206	47.6%	13.0%	0.000923247
T cell zone	LUM	7.24E-08	0.489000008	31.0%	4.7%	0.001092329
T cell zone	MYL9	7.38E-08	0.567871399	42.9%	9.8%	0.00111324
T cell zone	CALD1	1.88E-07	0.431730973	26.2%	3.1%	0.002844295
T cell zone	TRAJ17	2.61E-07	0.478326246	45.2%	11.9%	0.003932527
T cell zone	GREM1	2.78E-07	0.732634695	40.5%	9.8%	0.004200121
T cell zone	CCR7	3.04E-07	0.492780482	35.7%	7.3%	0.004588518
T cell zone	HLA-E	3.79E-07	0.632090497	88.1%	64.2%	0.005727426
T cell zone	CD3D	3.92E-07	0.727386617	64.3%	28.0%	0.005910034
T cell zone	COL3A1	6.17E-07	0.88427752	81.0%	44.0%	0.009311932
T cell zone	CRIM1	7.06E-07	0.449499083	35.7%	7.8%	0.010652566
T cell zone	GIMAP6	7.08E-07	0.250513137	21.4%	2.1%	0.010689154
T cell zone	B2M	8.05E-07	0.681479484	100.0%	86.5%	0.01215192
T cell zone	FLNA	9.91E-07	0.785102115	81.0%	60.6%	0.014957624
T cell zone	ITK	1.30E-06	0.407068918	26.2%	4.1%	0.019643798
T cell zone	CXCL12	1.47E-06	0.448818727	26.2%	4.1%	0.02219792
T cell zone	AEBP1	1.60E-06	0.356442845	26.2%	4.1%	0.024168985
T cell zone	CD96	1.80E-06	0.392784692	28.6%	5.2%	0.027104657
T cell zone	TRAJ36	1.99E-06	0.409235213	40.5%	10.9%	0.02998668
T cell zone	COL14A1	2.03E-06	0.47767359	21.4%	2.6%	0.030629117
T cell zone	DDX17	2.21E-06	0.53509579	97.6%	75.6%	0.033357613
T cell zone	TRAJ5	2.26E-06	0.519418131	40.5%	11.9%	0.034139031
T cell zone	SAMD9L	2.34E-06	0.346529101	31.0%	6.2%	0.035278182
T cell zone	TRIM22	2.55E-06	0.52318133	61.9%	27.5%	0.038442839
T cell zone	C1R	4.44E-06	0.691883287	61.9%	30.1%	0.066949422
T cell zone	C11orf96	4.51E-06	0.424571181	26.2%	4.7%	0.06807547
T cell zone	GIMAP4	4.56E-06	0.368537146	33.3%	7.8%	0.068778308
T cell zone	THBS1	5.51E-06	0.615177114	40.5%	13.0%	0.083211692
T cell zone	SPOCK2	6.23E-06	0.422532036	35.7%	9.3%	0.093965532
T cell zone	IL32	7.39E-06	0.679129747	71.4%	38.9%	0.111513473
T cell zone	LAMP3	1.26E-05	0.509824114	31.0%	7.3%	0.189764801
T cell zone	DUSP1	1.30E-05	0.41164372	42.9%	13.5%	0.196444351
T cell zone	TMEM176B	1.31E-05	0.350761478	31.0%	7.3%	0.198117713
T cell zone	TRBC2	1.35E-05	0.683081178	52.4%	23.3%	0.203347852
T cell zone	TMEM185A	1.51E-05	0.296815634	23.8%	4.1%	0.228570447
T cell zone	TRBC1	1.82E-05	0.829301919	69.0%	47.7%	0.274242617
T cell zone	DCN	2.08E-05	0.706325002	57.1%	26.9%	0.314041055
T cell zone	COL1A1	2.28E-05	0.883993872	47.6%	21.2%	0.344404816
T cell zone	EGR1	2.34E-05	0.467619925	35.7%	10.9%	0.353774134
T cell zone	TRAJ11	2.37E-05	0.341315677	33.3%	8.8%	0.35712784
T cell zone	PGGHG	2.58E-05	0.50784307	59.5%	28.0%	0.389544036
T cell zone	NLRP1	2.75E-05	0.300135404	31.0%	7.8%	0.414762065

T cell zone	IL6R	2.84E-05	0.334589814	35.7%	10.4%	0.42862076
T cell zone	NELL2	3.06E-05	0.353549529	23.8%	4.7%	0.461922193
T cell zone	ZFP36L2	3.18E-05	0.669015265	54.8%	27.5%	0.480136853
T cell zone	TBCK	3.80E-05	0.28105961	28.6%	6.7%	0.572859078
T cell zone	CHD2	4.03E-05	0.327895162	33.3%	9.3%	0.607493591
T cell zone	FOS	4.08E-05	0.57699392	52.4%	23.3%	0.615558397
T cell zone	DEPP1	4.46E-05	0.255634727	23.8%	4.7%	0.672716364
T cell zone	CD28	5.23E-05	0.483073578	45.2%	17.6%	0.789004568
T cell zone	FXYD6	5.93E-05	0.396059824	35.7%	11.4%	0.895160102
T cell zone	GIMAP5	6.73E-05	0.316163411	23.8%	5.2%	1
T cell zone	STX2	7.67E-05	0.256723143	19.0%	3.1%	1
T cell zone	TRAJ4	8.15E-05	0.294998801	26.2%	6.2%	1
T cell zone	TNKS1BP1	8.23E-05	0.268526589	26.2%	6.2%	1
T cell zone	VWF	8.31E-05	0.385695268	21.4%	4.1%	1
T cell zone	PLCB2	8.59E-05	0.316163411	21.4%	4.1%	1
T cell zone	C3	0.000105552	0.79678392	61.9%	37.3%	1
T cell zone	SPPL2B	0.000114219	0.300135404	28.6%	7.8%	1
T cell zone	DDIT4	0.000127536	0.514483114	54.8%	25.9%	1
T cell zone	FSTL1	0.000144827	0.307054818	26.2%	6.7%	1
T cell zone	FOXP1	0.000146003	0.494664306	59.5%	33.2%	1
T cell zone	TBC1D9	0.000153711	0.331929487	40.5%	15.0%	1
T cell zone	TSC22D3	0.000175225	0.386552739	40.5%	15.5%	1
T cell zone	CYTH4	0.000186866	0.300135404	28.6%	8.3%	1
T cell zone	NOP53	0.000190443	0.586980222	88.1%	74.6%	1
T cell zone	CMTM3	0.000198726	0.268526589	23.8%	5.7%	1
T cell zone	LTA4H	0.000202683	0.269848586	21.4%	4.7%	1
T cell zone	TRAJ3	0.000203502	0.262724152	21.4%	4.7%	1
T cell zone	PTGDS	0.000203752	0.73517689	92.9%	69.4%	1
T cell zone	MPEG1	0.000218767	0.488644708	52.4%	25.4%	1
T cell zone	GPATCH2L	0.000223885	0.336461086	38.1%	14.0%	1
T cell zone	TRAJ28	0.000234787	0.429226688	31.0%	10.4%	1
T cell zone	S100A4	0.000240579	0.435767863	45.2%	19.7%	1
T cell zone	ACTA2	0.000273646	0.626672753	45.2%	21.8%	1
T cell zone	CXCR4	0.000303774	0.557529455	83.3%	67.9%	1
T cell zone	STOM	0.000359021	0.327787438	38.1%	14.5%	1
T cell zone	FYB1	0.000402006	0.318784111	26.2%	7.8%	1
T cell zone	CTR9	0.000412507	0.303975425	19.0%	4.1%	1
T cell zone	ZFP36	0.000445649	0.516068969	42.9%	20.7%	1
T cell zone	CD247	0.000449145	0.303975425	19.0%	4.1%	1
T cell zone	TNXB	0.000449215	0.284203879	16.7%	3.1%	1
T cell zone	CD44	0.000460978	0.548775888	57.1%	32.1%	1
T cell zone	RAB37	0.000495088	0.26602755	31.0%	10.4%	1
T cell zone	CCN1	0.000524565	0.63044387	50.0%	27.5%	1
T cell zone	LAMA4	0.000541063	0.325670497	31.0%	10.9%	1
T cell zone	SH3TC1	0.000553106	0.31193044	28.6%	9.3%	1
T cell zone	OAS2	0.000591713	0.338845885	33.3%	12.4%	1
T cell zone	ARHGEF1	0.000687807	0.556283425	50.0%	31.1%	1
T cell zone	TRAJ13	0.000694647	0.328014437	21.4%	5.7%	1
T cell zone	DES	0.000766925	0.332975297	11.9%	1.6%	1
T cell zone	PCOLCE	0.000767404	0.280481723	35.7%	13.5%	1
T cell zone	ZNF609	0.000817014	0.314598339	31.0%	10.9%	1
T cell zone	DDX5	0.000906137	0.398602019	90.5%	77.2%	1
T cell zone	CBX6	0.000964387	0.284836779	28.6%	9.8%	1
T cell zone	COL1A2	0.001150294	0.959741405	69.0%	53.9%	1
T cell zone	TCF7	0.00139916	0.307995604	31.0%	11.9%	1
T cell zone	SKAP1	0.001465718	0.395331375	54.8%	31.1%	1
T cell zone	ITM2B	0.001509526	0.415198841	90.5%	64.8%	1
T cell zone	TRAJ16	0.001866949	0.299675288	31.0%	11.9%	1
T cell zone	MED13L	0.001897521	0.365063935	57.1%	33.2%	1
T cell zone	VAMP5	0.001933605	0.251669915	31.0%	11.9%	1
T cell zone	CTC1	0.002099315	0.294880104	31.0%	12.4%	1
T cell zone	FOSB	0.002113149	0.401519502	38.1%	17.1%	1
T cell zone	CD3E	0.002131911	0.36029684	40.5%	19.2%	1

T cell zone	CAVIN1	0.002140918	0.37598945	26.2%	9.8%	1
T cell zone	PBXIP1	0.002321477	0.464872308	54.8%	34.7%	1
T cell zone	TRAJ22	0.002405065	0.313233393	33.3%	14.0%	1
T cell zone	CCDC50	0.002421952	0.329977052	33.3%	14.5%	1
T cell zone	TRAJ24	0.002456159	0.284836779	26.2%	9.3%	1
T cell zone	ARHGAP22	0.00261326	0.293249019	21.4%	6.7%	1
T cell zone	CST3	0.002626818	0.327518921	45.2%	22.3%	1
T cell zone	COL4A1	0.002650634	0.343875092	21.4%	6.7%	1
T cell zone	TRAJ23	0.002741087	0.361890684	38.1%	18.7%	1
T cell zone	FLT3LG	0.00292106	0.294880104	31.0%	13.0%	1
T cell zone	MYLK	0.003064216	0.361683475	23.8%	8.8%	1
T cell zone	HLA-A	0.003120993	0.417446747	78.6%	60.6%	1
T cell zone	HABP4	0.003173903	0.265592676	35.7%	15.5%	1
T cell zone	NDUFA3	0.003205592	0.284836779	28.6%	11.4%	1
T cell zone	TRAJ27	0.003227455	0.392784692	28.6%	11.9%	1
T cell zone	TOMM7	0.003503017	0.360993626	73.8%	61.1%	1
T cell zone	REXO1	0.003896916	0.258242569	31.0%	13.0%	1
T cell zone	MSL1	0.004087458	0.359338209	40.5%	21.2%	1
T cell zone	FKBP8	0.004330339	0.364770316	69.0%	57.0%	1
T cell zone	MARCH6	0.004930722	0.342879787	45.2%	26.9%	1
T cell zone	NDRG2	0.00551513	0.293249019	31.0%	13.5%	1
T cell zone	WDR6	0.005713317	0.325670497	42.9%	23.8%	1
T cell zone	AHNAK	0.005789354	0.32352203	28.6%	12.4%	1
T cell zone	SRSF5	0.005825796	0.359522238	69.0%	50.3%	1
T cell zone	GRASP	0.005866781	0.330200155	23.8%	9.3%	1
T cell zone	NR4A3	0.006216232	0.298703449	11.9%	2.6%	1
T cell zone	SOD2	0.006616175	0.365361997	38.1%	19.7%	1
T cell zone	CHSY1	0.006907716	0.289936285	26.2%	10.9%	1
T cell zone	IFITM2	0.00698363	0.271692876	33.3%	15.5%	1
T cell zone	TRIM11	0.007002799	0.260400139	23.8%	9.3%	1
T cell zone	ATXN7	0.007096151	0.250765688	31.0%	14.0%	1
T cell zone	RNF145	0.007213377	0.366149566	40.5%	22.3%	1
T cell zone	YIPF3	0.007357248	0.333078446	45.2%	26.9%	1
T cell zone	ZYX	0.007601785	0.473900427	50.0%	32.1%	1
T cell zone	RNF149	0.00797162	0.287250278	40.5%	21.8%	1
T cell zone	TRAJ10	0.007981604	0.261540159	33.3%	16.1%	1
T cell zone	PFDN5	0.008122154	0.544094016	59.5%	45.6%	1
T cell zone	H1FX	0.008849553	0.403392792	47.6%	30.6%	1
T cell zone	COL6A1	0.009225674	0.374169014	33.3%	17.1%	1
T cell zone	PLEKHO1	0.009249649	0.333689945	57.1%	39.4%	1
T cell zone	CCL5	0.009254462	0.264845304	26.2%	11.4%	1
T cell zone	TGOLN2	0.009962096	0.255537853	54.8%	32.6%	1
SED/Epithelium	CLDN4	8.90E-19	1.15549208	80.0%	13.5%	1.34E-14
SED/Epithelium	CCL23	2.08E-18	1.129142136	54.3%	4.0%	3.13E-14
SED/Epithelium	KRT8	3.45E-16	1.652076697	85.7%	28.5%	5.21E-12
SED/Epithelium	IGKV4-1	5.44E-15	1.412211455	54.3%	6.0%	8.20E-11
SED/Epithelium	LGALS4	4.13E-14	1.009337865	80.0%	19.0%	6.23E-10
SED/Epithelium	CLDN7	9.09E-14	0.988745796	65.7%	12.5%	1.37E-09
SED/Epithelium	GOLM1	4.02E-13	1.024522319	71.4%	16.5%	6.07E-09
SED/Epithelium	FABP1	4.05E-13	0.958875267	65.7%	13.0%	6.11E-09
SED/Epithelium	MUC13	5.02E-13	0.64232872	51.4%	6.5%	7.57E-09
SED/Epithelium	NBL1	8.64E-13	0.596316322	60.0%	10.0%	1.30E-08
SED/Epithelium	PHGR1	1.16E-12	1.206450877	74.3%	20.5%	1.76E-08
SED/Epithelium	LAD1	1.95E-12	0.437330174	42.9%	4.0%	2.95E-08
SED/Epithelium	ATP1B1	2.04E-12	0.821754682	62.9%	12.0%	3.08E-08
SED/Epithelium	CCL15	2.32E-12	0.68985709	40.0%	3.5%	3.50E-08
SED/Epithelium	S100A6	2.82E-12	1.399095955	100.0%	73.5%	4.25E-08
SED/Epithelium	ITM2C	7.35E-12	1.140894703	85.7%	31.0%	1.11E-07
SED/Epithelium	AQP8	7.80E-12	0.5544659	42.9%	4.5%	1.18E-07
SED/Epithelium	JCHAIN	3.92E-11	1.823204445	94.3%	53.5%	5.91E-07
SED/Epithelium	LYPD8	6.36E-11	0.5898613	54.3%	9.5%	9.60E-07
SED/Epithelium	EPCAM	7.88E-11	0.830502527	62.9%	15.5%	1.19E-06
SED/Epithelium	TSPAN1	9.03E-11	0.642220996	54.3%	10.0%	1.36E-06

SED/Epithelium	EPS8L3	2.77E-10	0.315793309	22.9%	0.5%	4.18E-06
SED/Epithelium	PIGR	3.14E-10	0.830502527	62.9%	16.0%	4.74E-06
SED/Epithelium	APP	4.78E-10	1.005961558	71.4%	23.0%	7.21E-06
SED/Epithelium	CDH17	4.85E-10	0.600828906	40.0%	5.5%	7.32E-06
SED/Epithelium	KIAA1522	5.08E-10	0.351634602	31.4%	2.5%	7.66E-06
SED/Epithelium	IGKC	5.53E-10	1.880844845	100.0%	98.5%	8.34E-06
SED/Epithelium	KRT20	7.11E-10	0.599270337	42.9%	6.5%	1.07E-05
SED/Epithelium	TYROBP	1.15E-09	0.558967292	71.4%	19.0%	1.74E-05
SED/Epithelium	HNF4A	1.23E-09	0.32694617	28.6%	2.0%	1.85E-05
SED/Epithelium	TSPAN8	1.26E-09	0.468560775	48.6%	8.5%	1.91E-05
SED/Epithelium	MALL	2.88E-09	0.444183845	37.1%	5.0%	4.34E-05
SED/Epithelium	BHLHE41	3.78E-09	0.282626445	22.9%	1.0%	5.71E-05
SED/Epithelium	PTPRF	4.30E-09	0.592575685	42.9%	7.5%	6.49E-05
SED/Epithelium	SLC26A3	4.54E-09	0.940708381	65.7%	17.5%	6.85E-05
SED/Epithelium	TCF7L2	4.58E-09	0.571654268	48.6%	10.0%	6.91E-05
SED/Epithelium	EPB41L4B	5.04E-09	0.584651609	45.7%	8.5%	7.61E-05
SED/Epithelium	S100A14	8.31E-09	0.857874919	65.7%	21.5%	0.000125412
SED/Epithelium	CST3	9.26E-09	0.693170476	65.7%	19.5%	0.000139776
SED/Epithelium	LYZ	9.32E-09	1.317127109	82.9%	41.0%	0.000140681
SED/Epithelium	GUCA2A	9.70E-09	0.406048716	31.4%	3.5%	0.000146437
SED/Epithelium	IL1B	1.09E-08	0.344648171	31.4%	3.5%	0.000164285
SED/Epithelium	XBP1	1.12E-08	1.027307346	80.0%	36.5%	0.000169485
SED/Epithelium	GRN	1.15E-08	0.527064117	51.4%	11.5%	0.000172971
SED/Epithelium	ALDOB	1.30E-08	0.396736683	25.7%	2.0%	0.000196954
SED/Epithelium	KRT18	1.36E-08	0.452288895	37.1%	5.5%	0.000204908
SED/Epithelium	PDZK1IP1	1.52E-08	0.334000927	25.7%	2.0%	0.000229569
SED/Epithelium	AGPAT2	1.52E-08	0.334000927	25.7%	2.0%	0.000229569
SED/Epithelium	PPARG	1.64E-08	0.301579449	25.7%	2.0%	0.000247559
SED/Epithelium	SLC9A3	2.72E-08	0.710297644	54.3%	14.0%	0.000410025
SED/Epithelium	SOD3	2.79E-08	0.527064117	48.6%	10.5%	0.000420896
SED/Epithelium	MYH14	2.97E-08	0.382661497	28.6%	3.0%	0.000447681
SED/Epithelium	SPINT1	2.97E-08	0.351634602	28.6%	3.0%	0.000447681
SED/Epithelium	ELF3	3.23E-08	0.64441061	42.9%	8.5%	0.00048822
SED/Epithelium	CLCA4	3.36E-08	0.444183845	31.4%	4.0%	0.000507311
SED/Epithelium	HLA-DQB2	3.52E-08	0.308668874	22.9%	1.5%	0.000531239
SED/Epithelium	LMNA	4.10E-08	0.758383011	71.4%	26.0%	0.000618482
SED/Epithelium	GNA11	4.33E-08	0.337695411	31.4%	4.0%	0.000653502
SED/Epithelium	CEACAM1	4.82E-08	0.587329515	54.3%	14.5%	0.000728132
SED/Epithelium	SLC8A1	5.24E-08	0.261357828	22.9%	1.5%	0.000790709
SED/Epithelium	TMEM59	5.51E-08	0.543029619	65.7%	20.5%	0.00083152
SED/Epithelium	CLDN3	5.57E-08	0.371615219	34.3%	5.0%	0.000840171
SED/Epithelium	SLC39A4	6.83E-08	0.282626445	20.0%	1.0%	0.001031558
SED/Epithelium	PTK6	9.39E-08	0.294524692	25.7%	2.5%	0.001417805
SED/Epithelium	CCL20	9.98E-08	0.562101543	28.6%	3.5%	0.001506708
SED/Epithelium	COL6A2	1.06E-07	0.589760669	57.1%	16.5%	0.001599701
SED/Epithelium	CXCL14	1.09E-07	0.718401989	60.0%	18.5%	0.001639402
SED/Epithelium	GPRC5A	1.27E-07	0.457989644	28.6%	3.5%	0.001910683
SED/Epithelium	TRIM31	1.32E-07	0.415037499	28.6%	3.5%	0.001988406
SED/Epithelium	DNASE1L3	1.44E-07	0.548788888	51.4%	12.5%	0.002175094
SED/Epithelium	FERMT1	1.46E-07	0.337695411	28.6%	3.5%	0.002206024
SED/Epithelium	SELENBP1	1.50E-07	0.330775997	31.4%	4.5%	0.00225881
SED/Epithelium	FXYD3	1.74E-07	0.811290705	57.1%	18.0%	0.002621647
SED/Epithelium	ZG16	1.91E-07	0.619042439	54.3%	15.5%	0.002887908
SED/Epithelium	C1R	2.16E-07	0.707218251	74.3%	29.0%	0.003253391
SED/Epithelium	DMTN	2.51E-07	0.268412586	22.9%	2.0%	0.003793648
SED/Epithelium	ANPEP	2.59E-07	0.836501268	48.6%	14.0%	0.003916348
SED/Epithelium	TYMP	2.72E-07	0.461133914	40.0%	8.5%	0.004103705
SED/Epithelium	DDR1	2.91E-07	0.533681996	45.7%	11.5%	0.004393472
SED/Epithelium	PLS1	2.93E-07	0.261357828	22.9%	2.0%	0.004422643
SED/Epithelium	IGHA1	3.07E-07	1.757246625	88.6%	76.0%	0.004633686
SED/Epithelium	CDH1	3.24E-07	0.41881419	40.0%	8.5%	0.004893714
SED/Epithelium	BMPR2	3.50E-07	0.351634602	25.7%	3.0%	0.005283173
SED/Epithelium	KLF4	3.51E-07	0.666576266	60.0%	20.0%	0.005300558

SED/Epithelium	ITGAX	4.42E-07	0.610788488	48.6%	14.0%	0.006674815
SED/Epithelium	C19orf33	4.63E-07	0.378436485	31.4%	5.0%	0.006990431
SED/Epithelium	SDC4	5.23E-07	0.453461659	37.1%	7.5%	0.007888177
SED/Epithelium	MUC12	5.41E-07	0.584651609	40.0%	9.5%	0.008158879
SED/Epithelium	MICAL2	5.59E-07	0.475753924	45.7%	12.0%	0.008433069
SED/Epithelium	VSIR	5.59E-07	0.334452404	31.4%	5.0%	0.008443019
SED/Epithelium	F3	5.77E-07	0.27550201	20.0%	1.5%	0.008704331
SED/Epithelium	GPX2	5.77E-07	0.27550201	20.0%	1.5%	0.008704331
SED/Epithelium	OAF	5.77E-07	0.299067137	28.6%	4.0%	0.008708588
SED/Epithelium	MUC4	6.08E-07	0.31703594	28.6%	4.0%	0.009179227
SED/Epithelium	PRAP1	6.33E-07	0.310214674	31.4%	5.0%	0.009547105
SED/Epithelium	IGHG2	6.44E-07	2.017198492	80.0%	50.0%	0.009720819
SED/Epithelium	TIMP1	7.36E-07	0.502398459	51.4%	15.5%	0.01110191
SED/Epithelium	CEACAM5	8.12E-07	0.381090167	34.3%	6.5%	0.01226038
SED/Epithelium	LGR4	1.10E-06	0.378436485	31.4%	5.5%	0.016558444
SED/Epithelium	NCOA7	1.25E-06	0.467267458	57.1%	18.5%	0.018867037
SED/Epithelium	SMPDL3A	1.26E-06	0.261357828	22.9%	2.5%	0.018973631
SED/Epithelium	EGLN3	1.26E-06	0.261357828	22.9%	2.5%	0.018973631
SED/Epithelium	TRIM2	1.33E-06	0.287504264	22.9%	2.5%	0.020076792
SED/Epithelium	ANTXR1	1.36E-06	0.330775997	28.6%	4.5%	0.020505793
SED/Epithelium	HOXB6	1.45E-06	0.305986551	22.9%	2.5%	0.021942059
SED/Epithelium	ASAP2	1.54E-06	0.299067137	28.6%	4.5%	0.023222767
SED/Epithelium	AQP1	1.54E-06	0.299067137	28.6%	4.5%	0.023222767
SED/Epithelium	RALB	1.59E-06	0.280517834	25.7%	3.5%	0.02396507
SED/Epithelium	CYP3A5	1.65E-06	0.305986551	25.7%	3.5%	0.024876497
SED/Epithelium	CDHR5	1.66E-06	0.61747308	57.1%	20.5%	0.025088738
SED/Epithelium	IFI27	1.82E-06	0.407657969	42.9%	11.0%	0.027424911
SED/Epithelium	LAMC2	1.83E-06	0.273565073	25.7%	3.5%	0.027618284
SED/Epithelium	ST5	1.83E-06	0.273565073	25.7%	3.5%	0.027618284
SED/Epithelium	PRSS23	1.83E-06	0.273565073	25.7%	3.5%	0.027618284
SED/Epithelium	SDCBP2	1.83E-06	0.273565073	25.7%	3.5%	0.027618284
SED/Epithelium	LGALS3	1.97E-06	0.489038081	42.9%	11.5%	0.029664689
SED/Epithelium	RALGDS	2.08E-06	0.347923303	40.0%	9.5%	0.031334976
SED/Epithelium	SDC1	2.11E-06	0.266645659	25.7%	3.5%	0.03178769
SED/Epithelium	GRINA	2.31E-06	0.562401698	62.9%	25.5%	0.034848631
SED/Epithelium	IGHJ2	2.80E-06	0.501973136	40.0%	10.0%	0.042192351
SED/Epithelium	CDX2	3.29E-06	0.268412586	20.0%	2.0%	0.049716132
SED/Epithelium	PPP1R14C	3.44E-06	0.410236513	34.3%	7.5%	0.051850697
SED/Epithelium	UTS2B	3.65E-06	0.351342824	31.4%	6.0%	0.055112087
SED/Epithelium	CD63	3.68E-06	0.638444831	65.7%	32.0%	0.055548684
SED/Epithelium	GALNT2	3.82E-06	0.434573851	51.4%	16.5%	0.057641862
SED/Epithelium	SQSTM1	3.89E-06	0.646971708	91.4%	80.0%	0.058753577
SED/Epithelium	LRP10	3.92E-06	0.499413366	74.3%	32.5%	0.059179025
SED/Epithelium	LGALS3BP	4.00E-06	0.608965543	65.7%	27.5%	0.060432209
SED/Epithelium	IER5	4.22E-06	0.415037499	51.4%	16.5%	0.063731004
SED/Epithelium	NR3C2	4.30E-06	0.31703594	28.6%	5.0%	0.064967203
SED/Epithelium	CDC42EP5	4.43E-06	0.443019912	51.4%	16.0%	0.066889399
SED/Epithelium	IGHG3	4.55E-06	1.420108489	54.3%	19.5%	0.068657077
SED/Epithelium	AGAP1	4.90E-06	0.28532708	28.6%	5.0%	0.073961177
SED/Epithelium	TMEM273	4.98E-06	0.2543374	22.9%	3.0%	0.075161527
SED/Epithelium	RNF207	5.06E-06	0.273565073	25.7%	4.0%	0.076440924
SED/Epithelium	IL22RA2	5.09E-06	0.327695038	28.6%	5.0%	0.076889709
SED/Epithelium	HMGCS2	5.16E-06	0.280517834	22.9%	3.0%	0.077942616
SED/Epithelium	SPINT2	5.23E-06	0.409603613	42.9%	12.0%	0.0789518
SED/Epithelium	ATP8B1	5.26E-06	0.482724307	40.0%	10.5%	0.07939738
SED/Epithelium	IGLV3-1	5.27E-06	0.955842214	34.3%	8.0%	0.079601136
SED/Epithelium	S100A11	5.44E-06	0.453690931	51.4%	17.0%	0.082071179
SED/Epithelium	RGS1	5.47E-06	0.305119807	37.1%	8.5%	0.082514214
SED/Epithelium	CSF1R	5.62E-06	0.370526803	37.1%	9.0%	0.084806045
SED/Epithelium	SLC39A5	5.81E-06	0.31703594	25.7%	4.0%	0.087690543
SED/Epithelium	OS9	6.04E-06	0.64546724	82.9%	47.5%	0.091200133
SED/Epithelium	NOL4L	6.39E-06	0.334452404	31.4%	6.5%	0.096385886
SED/Epithelium	FTH1	6.60E-06	0.57564875	100.0%	93.5%	0.09963317

SED/Epithelium	TMBIM1	7.25E-06	0.461133914	37.1%	9.5%	0.109373129
SED/Epithelium	SELENOP	7.79E-06	0.384512632	45.7%	13.5%	0.117646657
SED/Epithelium	OLFM1	8.12E-06	0.435138705	40.0%	11.0%	0.122605734
SED/Epithelium	TPM1	8.77E-06	0.607682577	82.9%	40.5%	0.132392457
SED/Epithelium	LRATD1	1.00E-05	0.501749132	40.0%	11.5%	0.151013486
SED/Epithelium	S100A10	1.01E-05	0.793727244	77.1%	54.0%	0.152361529
SED/Epithelium	FHL2	1.13E-05	0.324748614	34.3%	8.0%	0.169924214
SED/Epithelium	PLAUR	1.21E-05	0.482724307	42.9%	12.5%	0.183350551
SED/Epithelium	CTSD	1.23E-05	0.634867407	91.4%	70.5%	0.186385637
SED/Epithelium	PPP2CB	1.24E-05	0.474495733	71.4%	32.0%	0.187873289
SED/Epithelium	GSN	1.25E-05	0.560376862	80.0%	41.0%	0.188081073
SED/Epithelium	RNF130	1.26E-05	0.299067137	25.7%	4.5%	0.190144571
SED/Epithelium	ITGA3	1.39E-05	0.348062836	25.7%	4.5%	0.210394184
SED/Epithelium	AGRN	1.40E-05	0.261357828	20.0%	2.5%	0.211820201
SED/Epithelium	CMBL	1.57E-05	0.2543374	20.0%	2.5%	0.23740773
SED/Epithelium	TCIM	1.62E-05	0.335249473	42.9%	12.5%	0.243917219
SED/Epithelium	SATB2	1.71E-05	0.280517834	20.0%	2.5%	0.257508655
SED/Epithelium	SLC44A4	1.72E-05	0.445531528	28.6%	6.0%	0.259410206
SED/Epithelium	VAT1L	1.81E-05	0.252905603	25.7%	4.5%	0.272888107
SED/Epithelium	RAB25	1.88E-05	0.351074441	31.4%	7.0%	0.284447797
SED/Epithelium	COL3A1	1.93E-05	0.518184426	82.9%	45.0%	0.291695923
SED/Epithelium	POR	1.97E-05	0.413289837	45.7%	15.0%	0.297074574
SED/Epithelium	ARRDC4	2.19E-05	0.344648171	40.0%	11.5%	0.331262336
SED/Epithelium	BRI3	2.29E-05	0.414222647	54.3%	19.5%	0.345189808
SED/Epithelium	CTSS	2.33E-05	0.509355464	65.7%	30.0%	0.351332639
SED/Epithelium	ID1	2.33E-05	0.358068687	28.6%	6.0%	0.352219189
SED/Epithelium	CD68	2.38E-05	0.427110332	48.6%	17.5%	0.359004173
SED/Epithelium	TMEM54	2.45E-05	0.320969175	28.6%	6.0%	0.369318738
SED/Epithelium	GPX4	2.60E-05	0.468279521	68.6%	32.0%	0.392819901
SED/Epithelium	LLGL2	2.71E-05	0.344648171	31.4%	7.5%	0.409757146
SED/Epithelium	S100A16	2.78E-05	0.323889611	25.7%	5.0%	0.418925381
SED/Epithelium	A2M	2.81E-05	0.320969175	31.4%	7.5%	0.424543864
SED/Epithelium	IGHG4	2.90E-05	0.906619029	31.4%	7.5%	0.437717223
SED/Epithelium	SBNO2	3.01E-05	0.335249473	42.9%	13.0%	0.454677009
SED/Epithelium	DSC2	3.14E-05	0.292180751	25.7%	5.0%	0.473521365
SED/Epithelium	SH3BP2	3.52E-05	0.28532708	25.7%	5.0%	0.531256042
SED/Epithelium	IGLC1	3.54E-05	2.538871769	71.4%	55.5%	0.533670765
SED/Epithelium	IGFBP7	3.68E-05	0.488777172	88.6%	51.0%	0.55566267
SED/Epithelium	ITM2B	3.95E-05	0.606203648	82.9%	67.0%	0.59656839
SED/Epithelium	PLOD2	4.17E-05	0.305986551	22.9%	4.0%	0.628656971
SED/Epithelium	CCR1	4.19E-05	0.301579449	17.1%	2.0%	0.631752436
SED/Epithelium	FLNB	4.21E-05	0.276583896	31.4%	7.5%	0.636061571
SED/Epithelium	CCDC47	4.55E-05	0.375809103	40.0%	12.5%	0.687235501
SED/Epithelium	P2RX4	4.58E-05	0.303425509	28.6%	6.5%	0.691917258
SED/Epithelium	HLA-A	4.79E-05	0.569020957	88.6%	59.5%	0.723326266
SED/Epithelium	BHLHE40	5.19E-05	0.298635774	34.3%	9.0%	0.783864389
SED/Epithelium	CBLC	5.21E-05	0.367731785	28.6%	6.5%	0.786475967
SED/Epithelium	ABCC3	5.50E-05	0.397003576	40.0%	12.5%	0.829386725
SED/Epithelium	CA4	5.55E-05	0.337984441	28.6%	6.5%	0.837627034
SED/Epithelium	OBSCN	5.93E-05	0.264959283	28.6%	6.5%	0.894721449
SED/Epithelium	PDCD4	6.25E-05	0.41654109	62.9%	26.5%	0.943710217
SED/Epithelium	ORAI3	6.27E-05	0.31703594	25.7%	5.5%	0.946232805
SED/Epithelium	ISCU	7.00E-05	0.377069649	48.6%	18.0%	1
SED/Epithelium	MYO15B	7.32E-05	0.269950804	28.6%	6.5%	1
SED/Epithelium	CLTB	7.52E-05	0.491853096	54.3%	23.5%	1
SED/Epithelium	BCL3	7.85E-05	0.392943728	42.9%	14.5%	1
SED/Epithelium	SLC26A2	8.04E-05	0.305119807	34.3%	9.5%	1
SED/Epithelium	RNASET2	8.96E-05	0.494673615	65.7%	31.5%	1
SED/Epithelium	PLEC	9.05E-05	0.618681576	62.9%	32.0%	1
SED/Epithelium	C1QC	9.16E-05	0.450442835	37.1%	12.0%	1
SED/Epithelium	P4HB	9.18E-05	0.530340489	62.9%	29.0%	1
SED/Epithelium	SGK1	9.25E-05	0.399095955	42.9%	15.0%	1
SED/Epithelium	PHLDA1	0.000106666	0.283247627	25.7%	5.5%	1

SED/Epithelium	FCGRT	0.00010716	0.986641617	82.9%	63.5%	1
SED/Epithelium	PTPRE	0.000107947	0.275786313	42.9%	14.0%	1
SED/Epithelium	NDRG2	0.000115171	0.281912416	40.0%	12.5%	1
SED/Epithelium	MGAT4B	0.000120778	0.413289837	42.9%	15.5%	1
SED/Epithelium	HKDC1	0.000123879	0.280517834	20.0%	3.5%	1
SED/Epithelium	IGHG1	0.000149631	3.109810657	65.7%	42.5%	1
SED/Epithelium	SREBF1	0.000154324	0.310481568	42.9%	15.0%	1
SED/Epithelium	HSPB1	0.000161112	0.49220536	74.3%	43.0%	1
SED/Epithelium	PEX26	0.000164224	0.467609694	60.0%	29.0%	1
SED/Epithelium	COL6A3	0.000170424	0.265096872	34.3%	9.5%	1
SED/Epithelium	CLPTM1	0.000171788	0.310700839	54.3%	21.5%	1
SED/Epithelium	CTSC	0.000192469	0.266645659	37.1%	11.5%	1
SED/Epithelium	MYO7B	0.000212113	0.374642912	40.0%	14.0%	1
SED/Epithelium	MMP14	0.00021835	0.370526803	31.4%	9.5%	1
SED/Epithelium	ETHE1	0.000242387	0.261807102	34.3%	10.5%	1
SED/Epithelium	B2M	0.000256975	0.531061296	94.3%	88.0%	1
SED/Epithelium	C1QB	0.000262035	0.305119807	34.3%	11.0%	1
SED/Epithelium	MMP9	0.000277846	0.729796099	65.7%	37.5%	1
SED/Epithelium	GMDS	0.000280115	0.296668142	25.7%	6.5%	1
SED/Epithelium	AFDN	0.000294433	0.425568167	40.0%	15.0%	1
SED/Epithelium	PTGS1	0.000300978	0.370526803	31.4%	9.5%	1
SED/Epithelium	PPP1R1B	0.000301437	0.299067137	20.0%	4.0%	1
SED/Epithelium	PITX2	0.000301437	0.299067137	20.0%	4.0%	1
SED/Epithelium	MPEG1	0.000309701	0.3956321	57.1%	25.5%	1
SED/Epithelium	SQOR	0.000315054	0.509278873	54.3%	27.5%	1
SED/Epithelium	TGFBI	0.000319521	0.264959283	25.7%	6.5%	1
SED/Epithelium	CCDC50	0.00032065	0.288064643	40.0%	14.0%	1
SED/Epithelium	PLEKHG6	0.000320797	0.323889611	20.0%	4.0%	1
SED/Epithelium	PARP15	0.000330214	0.294126396	45.7%	16.5%	1
SED/Epithelium	CISD3	0.000339868	0.41147968	37.1%	13.5%	1
SED/Epithelium	PIK3IP1	0.000400209	0.274746158	31.4%	9.5%	1
SED/Epithelium	ANXA2	0.000412275	0.466210151	57.1%	28.5%	1
SED/Epithelium	IGHJ6	0.000419539	0.905487878	97.1%	92.0%	1
SED/Epithelium	IER3	0.00042052	0.615950193	45.7%	20.5%	1
SED/Epithelium	CCN2	0.000427845	0.429850494	48.6%	20.5%	1
SED/Epithelium	ZFP36L2	0.000428952	0.250553656	62.9%	27.0%	1
SED/Epithelium	ACTN4	0.000441269	0.302123555	45.7%	18.5%	1
SED/Epithelium	INF2	0.000445491	0.525220417	54.3%	27.0%	1
SED/Epithelium	TNFRSF21	0.000448119	0.311633114	31.4%	10.0%	1
SED/Epithelium	PLSCR1	0.00048132	0.49220536	48.6%	22.5%	1
SED/Epithelium	NR4A1	0.000499183	0.464820138	34.3%	12.0%	1
SED/Epithelium	CD44	0.000528663	0.388195887	62.9%	32.0%	1
SED/Epithelium	HLA-DPB1	0.000547209	0.439103928	88.6%	77.5%	1
SED/Epithelium	SIGLEC10	0.000556425	0.338495944	40.0%	15.5%	1
SED/Epithelium	TRPM4	0.000558581	0.283247627	25.7%	7.0%	1
SED/Epithelium	ITGAV	0.000583743	0.25823342	25.7%	7.0%	1
SED/Epithelium	AIF1	0.000583743	0.25823342	25.7%	7.0%	1
SED/Epithelium	FOSB	0.000604808	0.581687369	40.0%	17.5%	1
SED/Epithelium	SOCS3	0.000622715	0.274746158	28.6%	8.5%	1
SED/Epithelium	ACSL5	0.000630329	0.292180751	34.3%	12.0%	1
SED/Epithelium	GNPTG	0.000658044	0.31955719	51.4%	23.0%	1
SED/Epithelium	ST6GALNAC6	0.00066349	0.286131707	42.9%	17.0%	1
SED/Epithelium	C3	0.000684356	0.466210151	68.6%	37.0%	1
SED/Epithelium	HLA-F	0.000689164	0.364210438	62.9%	30.5%	1
SED/Epithelium	IGHM	0.000711074	0.573700478	94.3%	81.0%	1
SED/Epithelium	LTBP3	0.00072356	0.256261177	45.7%	18.0%	1
SED/Epithelium	NUMB	0.000728301	0.368593867	40.0%	15.0%	1
SED/Epithelium	STOM	0.000760496	0.257562555	40.0%	15.0%	1
SED/Epithelium	PLXNB2	0.000811485	0.394479327	68.6%	36.5%	1
SED/Epithelium	KDM2A	0.000831278	0.256261177	42.9%	17.0%	1
SED/Epithelium	C4orf48	0.000859252	0.309104055	34.3%	12.5%	1
SED/Epithelium	TNFRSF1A	0.000875755	0.314274522	25.7%	7.5%	1
SED/Epithelium	PTMS	0.000887766	0.339131166	51.4%	24.5%	1

SED/Epithelium	ICAM2	0.000899644	0.437757576	51.4%	26.0%	1
SED/Epithelium	ADGRE5	0.000913906	0.268262125	31.4%	10.5%	1
SED/Epithelium	TMEM173	0.000945375	0.30273453	34.3%	12.5%	1
SED/Epithelium	C15orf48	0.001000392	0.386098615	28.6%	9.0%	1
SED/Epithelium	KIF1C	0.001035322	0.279356711	34.3%	12.5%	1
SED/Epithelium	NFE2L1	0.001096829	0.324389332	48.6%	22.0%	1
SED/Epithelium	MAP7D1	0.001112763	0.278374952	42.9%	17.5%	1
SED/Epithelium	GANAB	0.001218891	0.299979122	45.7%	19.5%	1
SED/Epithelium	RXRA	0.001270732	0.277533976	34.3%	12.5%	1
SED/Epithelium	IGFBP3	0.001283118	0.372554168	45.7%	20.5%	1
SED/Epithelium	ARMC8	0.001297139	0.271302022	37.1%	14.5%	1
SED/Epithelium	EFHD2	0.001387445	0.390028075	54.3%	27.5%	1
SED/Epithelium	MFGE8	0.001408742	0.378200731	48.6%	22.5%	1
SED/Epithelium	CLCN3	0.001473555	0.300448367	37.1%	15.0%	1
SED/Epithelium	GDE1	0.001537602	0.264959283	22.9%	6.5%	1
SED/Epithelium	IFNGR2	0.001638511	0.323889611	88.6%	66.0%	1
SED/Epithelium	SLC5A1	0.001640442	0.264959283	20.0%	5.0%	1
SED/Epithelium	RAB5A	0.001654585	0.254706046	60.0%	29.5%	1
SED/Epithelium	AFAP1	0.001690758	0.251538767	22.9%	6.5%	1
SED/Epithelium	HMGN4	0.00188111	0.284705631	48.6%	22.0%	1
SED/Epithelium	MAPK13	0.001900866	0.255380833	31.4%	11.5%	1
SED/Epithelium	CSF2RB	0.001918587	0.299979122	42.9%	19.0%	1
SED/Epithelium	CD55	0.00196242	0.281259465	28.6%	10.0%	1
SED/Epithelium	CLCN7	0.002167975	0.258918297	37.1%	15.0%	1
SED/Epithelium	PKIG	0.002195014	0.303069068	51.4%	24.0%	1
SED/Epithelium	AKAP1	0.002284769	0.341736576	40.0%	17.5%	1
SED/Epithelium	CCN1	0.002345777	0.40693245	54.3%	27.5%	1
SED/Epithelium	ST14	0.002380017	0.317681124	40.0%	18.0%	1
SED/Epithelium	PLA2G2D	0.002435855	0.268135278	40.0%	17.0%	1
SED/Epithelium	ASAH1	0.00245134	0.347116427	57.1%	30.0%	1
SED/Epithelium	CAPN2	0.002469928	0.309104055	31.4%	12.0%	1
SED/Epithelium	STAT3	0.002500874	0.347367673	51.4%	26.0%	1
SED/Epithelium	OCIAD1	0.002579841	0.275107238	48.6%	23.0%	1
SED/Epithelium	SEC11A	0.002848509	0.297342457	57.1%	30.0%	1
SED/Epithelium	THEMIS2	0.002940545	0.360450028	31.4%	12.5%	1
SED/Epithelium	CTSA	0.003142391	0.250232569	28.6%	10.5%	1
SED/Epithelium	BSG	0.003146834	0.342022702	57.1%	30.0%	1
SED/Epithelium	EIF1	0.003254352	0.287037448	57.1%	30.5%	1
SED/Epithelium	PLPP1	0.003575736	0.287802311	25.7%	9.0%	1
SED/Epithelium	GATAD2B	0.003669283	0.25276607	37.1%	16.0%	1
SED/Epithelium	TSPAN13	0.003704632	0.277533976	34.3%	14.5%	1
SED/Epithelium	FOS	0.003719041	0.463226293	48.6%	25.0%	1
SED/Epithelium	JUN	0.003720925	0.32363039	37.1%	16.0%	1
SED/Epithelium	TXNDC5	0.003781279	0.367731785	22.9%	7.5%	1
SED/Epithelium	APLP2	0.003854703	0.288691765	51.4%	25.5%	1
SED/Epithelium	ANKRD10	0.003903166	0.274109179	40.0%	18.0%	1
SED/Epithelium	HBEGF	0.003908012	0.323889611	17.1%	4.5%	1
SED/Epithelium	CREB3L1	0.004103273	0.266645659	17.1%	4.5%	1
SED/Epithelium	GAPT	0.004215618	0.292180751	37.1%	16.0%	1
SED/Epithelium	MYADM	0.004252455	0.283792966	34.3%	15.0%	1
SED/Epithelium	HERPUD1	0.004373953	0.253448357	65.7%	35.5%	1
SED/Epithelium	ERN1	0.004455937	0.30273453	28.6%	11.0%	1
SED/Epithelium	SERPINF1	0.004547061	0.283792966	31.4%	13.0%	1
SED/Epithelium	TXNDC12	0.004660814	0.272079545	48.6%	24.0%	1
SED/Epithelium	LITAF	0.005431568	0.324389332	42.9%	21.0%	1
SED/Epithelium	CALCOCO2	0.00543888	0.269686114	34.3%	15.0%	1
SED/Epithelium	ADI1	0.00591833	0.312939312	42.9%	21.5%	1
SED/Epithelium	SYNGR2	0.006212117	0.368416337	57.1%	35.0%	1
SED/Epithelium	TM9SF3	0.006439814	0.268135278	37.1%	17.0%	1
SED/Epithelium	DHRS9	0.006489227	0.319225575	31.4%	13.5%	1
SED/Epithelium	CYC1	0.006513584	0.337268641	54.3%	31.5%	1
SED/Epithelium	COL6A1	0.006520347	0.294126396	37.1%	17.0%	1
SED/Epithelium	ECE1	0.006533098	0.324389332	40.0%	19.5%	1

SED/Epithelium	RHOF	0.007520661	0.313875823	62.9%	41.0%	1
SED/Epithelium	ERRFI1	0.007565162	0.250232569	25.7%	10.0%	1
SED/Epithelium	MAN2B2	0.007591818	0.271302022	31.4%	13.5%	1
SED/Epithelium	MIB2	0.007632382	0.302123555	37.1%	17.5%	1
SED/Epithelium	SCRIB	0.008275221	0.296149654	37.1%	18.0%	1
SED/Epithelium	IL16	0.008287435	0.257013897	48.6%	25.5%	1
SED/Epithelium	CRELD2	0.00861662	0.336786054	48.6%	27.5%	1
SED/Epithelium	RNF11	0.008742508	0.295738571	45.7%	25.0%	1
SED/Epithelium	LAMP1	0.008818023	0.312939312	42.9%	23.0%	1
SED/Epithelium	WDR45B	0.009586459	0.265096872	31.4%	14.0%	1
SED/Epithelium	SOD2	0.009770353	0.259500053	40.0%	20.0%	1
