# Supplementary material for: Double-negative B cells and DNASE1L3 colocalise with microbiota in gut-associated lymphoid tissue
Source: Nat Commun. 2024 May 14;15:4051. doi: 10.1038/s41467-024-48267-4 (PMC11094119; doi:10.1038/s41467-024-48267-4)
Supplement: Supplementary file 3 — Description of Additional Supplementary Files [file 41467_2024_48267_MOESM3_ESM.pdf]

Description of Additional Supplementary Files

Supplementary Movie 1

Video illustrating a volume-reconstruction of the confocal image in Fig. 8h, progressively showing the acquired Z-stacks from the top to the bottom of the section. Slides were stained with Bacterial 16S probe (green, pseudocolor), counterstained with DAPI (blue) and imaged as described in the method section.

Supplementary Movie 2

Video illustrating a volume-reconstruction of the confocal image in Fig. 8h and Suppl. Movie 1, showing the image from different angles.
