# Supplementary material for: Double-negative B cells and DNASE1L3 colocalise with microbiota in gut-associated lymphoid tissue
Source: Nat Commun. 2024 May 14;15:4051. doi: 10.1038/s41467-024-48267-4 (PMC11094119; doi:10.1038/s41467-024-48267-4)
Supplement: Supplementary file 6 — Reporting Summary [file 41467_2024_48267_MOESM6_ESM.pdf]

Corresponding author(s): Jo Spencer

Last updated by author(s): 12 APRIL 2024

Reporting Summary

Nature Portfolio wishes to improve the reproducibility of the work that we publish. This form provides structure for consistency and transparency in reporting. For further information on Nature Portfolio policies, see our Editorial Policies and the Editorial Policy Checklist.

Statistics

For all statistical analyses, confirm that the following items are present in the figure legend, table legend, main text, or Methods section.

n/a	Confirmed	
☐	☒	The exact sample size (*n*) for each experimental group/condition, given as a discrete number and unit of measurement
☐	☒	A statement on whether measurements were taken from distinct samples or whether the same sample was measured repeatedly
☐	☒	The statistical test(s) used AND whether they are one- or two-sided<br>*Only common tests should be described solely by name; describe more complex techniques in the Methods section.*
☐	☒	A description of all covariates tested
☐	☒	A description of any assumptions or corrections, such as tests of normality and adjustment for multiple comparisons
☐	☒	A full description of the statistical parameters including central tendency (e.g. means) or other basic estimates (e.g. regression coefficient) AND variation (e.g. standard deviation) or associated estimates of uncertainty (e.g. confidence intervals)
☐	☒	For null hypothesis testing, the test statistic (e.g. *F*, *t*, *r*) with confidence intervals, effect sizes, degrees of freedom and *P* value noted<br>*Give P values as exact values whenever suitable.*
☒	☐	For Bayesian analysis, information on the choice of priors and Markov chain Monte Carlo settings
☒	☐	For hierarchical and complex designs, identification of the appropriate level for tests and full reporting of outcomes
☒	☐	Estimates of effect sizes (e.g. Cohen's *d*, Pearson's *r*), indicating how they were calculated

Our web collection on statistics for biologists contains articles on many of the points above.

Software and code

Policy information about availability of computer code

Data collection	Code is not involved in data collection.
Data analysis	Code used to perform analyses is available at github.com/jspencer-lab/GALT_analysis_2023.<br>Package versions:<br><br>IMC<br>Python:<br>Conda environment from BodenmillerGroup/ImcSegmentationPipeline. See Usage at https://github.com/BodenmillerGroup/ImcSegmentationPipeline.<br>CellProfiler:<br>CellProfiler v4.<br>R:<br>cytomapper v1.14.0<br>imcRtools v1.8.0<br>harmony v1.1.0<br>randomForest 4.7-1.1<br>cowplot v1.1.1<br>bluster v1.12.0<br>viridis v0.6.4

CATALYST v1.26.0
dittoSeq v1.14.0
mclust v6.0.0
scran vv1.30.0
patchwork v1.1.3
caret v6.0-94
tidyverse v2.0.0
Hmisc v5.1-1
devtools v2.4.5
dichromat v2.0-0.1
Rphenograph v0.99.1.9003

CyTOF
Cytobank:
Pre-processing was done on Cytobank (https://mrc.cytobank.org) version 10.3
R:
R packages as per IMC

RNAScope
CellProfiler:
CellProfiler v4.
R:
cytomapper v1.12.0
stringr v1.5.0
imcRtools v1.6.4
dplyr v1.1.3
Biostrings v2.68.1
GenomicAlignments v1.36.0
scoper v1.3.0
scater v1.28.0
batchelor v1.16.0
Rphenograph v0.99.1.903
igraph v1.5.1
dittoSeq v1.12.1
tidyr v1.3.0
tibble v3.2.1
viridis 0.6.4
pheatmap v1.0.12

Visium
R:
dplyr v1.1.4
ggplot2 v3.4.4
patchwork v1.2.0
Seurat v4.4.0
viridis v0.6.4
BayesSpace v1.10.1
Harmony v0.1.1
ggpubr v0.6.0
RcmdrMisc v2.9-0
corrplot v0.92

For manuscripts utilizing custom algorithms or software that are central to the research but not yet described in published literature, software must be made available to editors and reviewers. We strongly encourage code deposition in a community repository (e.g. GitHub). See the Nature Portfolio guidelines for submitting code & software for further information.

Data

Policy information about availability of data

All manuscripts must include a data availability statement. This statement should provide the following information, where applicable:

- Accession codes, unique identifiers, or web links for publicly available datasets
- A description of any restrictions on data availability
- For clinical datasets or third party data, please ensure that the statement adheres to our policy

The spatial transcriptomic data generated in this study have been deposited in the GEO database under accession code GSE251693 [https://www.ncbi.nlm.nih.gov/geo/query/acc.cgi?acc=GSE251693]. The IMC image data generated in this study have been deposited on zenodo under record 10853309 [https://doi.org/10.5281/zenodo.10853309].

Research involving human participants, their data, or biological material

Policy information about studies with human participants or human data. See also policy information about sex, gender (identity/presentation), and sexual orientation and race, ethnicity and racism.

Reporting on sex and gender	Where sex and gender are known, these are included in the manuscript. Most of the tissues used for this
Reporting on race, ethnicity, or other socially relevant groupings	Information on ethnicity is not available for the tissues used in this study.
Population characteristics	All formalin fixed paraffin embedded tissues used in this study were anonymous or anonymised for researchers.<br><br>Gut biopsies and paired blood samples used for Mass Cytometry were obtained with informed consent and REC approval from 5 healthy patients undergoing investigative colonoscopy who had no evidence of inflammation or other pathology. Mononuclear cells from gut biopsies and blood, were isolated as described previously and were stained on day of collection. These were 3 males aged 56, 56, 72 and 2 females aged 30 and 61.
Recruitment	Patients were recruited by trained staff with informed concent and REC approval.
Ethics oversight	Our study 'Immunology of the intestine; features associated with autoimmunity. Number 11/LO/1274' was approved by London- Camberwell St Giles Research Ethics Committee.

Note that full information on the approval of the study protocol must also be provided in the manuscript.

Field-specific reporting

Please select the one below that is the best fit for your research. If you are not sure, read the appropriate sections before making your selection.

[x] Life sciences [ ] Behavioural & social sciences [ ] Ecological, evolutionary & environmental sciences

For a reference copy of the document with all sections, see nature.com/documents/nr-reporting-summary-flat.pdf

Life sciences study design

All studies must disclose on these points even when the disclosure is negative.

Sample size	Our manuscript describes features of human gut-associated lymphoid tissue (GALT). This tissue is dotted through the human gastrointestinal tract and it is not possible to select specific numbers of follicles of GALT in a predetermined way. In addition, our study required that GALT, which is highly polarised in terms of microanatomy and cell content, is present in the correct orientation for all cell types to be included in the analysis. We selected samples of GALT from 3 sites and acquired as much data as we could from each. Our study of such rare structures included sufficient replicates to achieve significant data sets that could not have been predicted at the start.
Data exclusions	Data was not used if GALT was not in the correct microanatomical orientation for the analysis once it was acquired.
Replication	It is not feasible to provide technical replicates for the techniques used in this study due to the following reasons:<br>-each tissues section is unique and it is therefore impossible to repeat the same histological stain multiple times- this applies to figure 2-8<br><br>-serial sections, which are the closest possible technical duplicates, were required for the study design to cross reference between data types<br><br>-because of this approach we have analysed different donors and various tissues for each donor and multiple ROIs for each tissue where possible to ensure reproducibility
Randomization	All tissues studied were normal and there was no grouping other than original of tissue.
Blinding	It was not possible to blind this study though much of the work uses undirected analysis methods which by their nature are blinded to the researcher.

Reporting for specific materials, systems and methods

We require information from authors about some types of materials, experimental systems and methods used in many studies. Here, indicate whether each material, system or method listed is relevant to your study. If you are not sure if a list item applies to your research, read the appropriate section before selecting a response.

Materials & experimental systems

n/a	Involved in the study
☐	☒ Antibodies
☒	☐ Eukaryotic cell lines
☒	☐ Palaeontology and archaeology
☐	☒ Animals and other organisms
☒	☐ Clinical data
☒	☐ Dual use research of concern
☒	☐ Plants

Methods

n/a	Involved in the study
☒	☐ ChIP-seq
☒	☐ Flow cytometry
☒	☐ MRI-based neuroimaging

Antibodies

Antibodies used

Antibodies used for Cytof (Supplementary Table 1)

Marker Metal Tag Clone Cat Number Supplier Dilution
BCMA 174Yb 19F2 357502 Biolegend 1:100
CCR10 (GPR-2) 166Er 314305 MAB3478 R&D Systems 1:100
CD10 158Gd HI10a 3158011B Fluidigm (now Standardbiotools) 1:100
CD138 150Nd DL-101 3150012B Fluidigm (now Standardbiotools) 1:100
CD180 176Yb MHR73-11 312902 Biolegend 1:100
CD19 165Ho HIB19 3165025B Fluidigm (now Standardbiotools) 1:100
CD196 (CCR6) 141Pr 11A9 3141014A Fluidigm (now Standardbiotools) 1:100
CD197 (CCR7) 175Lu G043H7 353202 Biolegend 1:100
CD20 147Sm 2H7 3147001B Fluidigm (now Standardbiotools) 1:100
CD21 152Sm BL13 3152010B Fluidigm (now Standardbiotools) 1:100
CD22 159Tb HIB22 3159005B Fluidigm (now Standardbiotools) 1:100
CD23 164Dy EBVCS-5 3164018B Fluidigm (now Standardbiotools) 1:100
CD24 169Tm ML5 3169004B Fluidigm (now Standardbiotools) 1:100
CD268 (BAFF-R) 155Gd 11C1 3155005B Fluidigm (now Standardbiotools) 1:100
CD27 167Er O323 3167002B Fluidigm (now Standardbiotools) 1:100
CD29 (intergrin b1) 156Gd TS2/16 3156007B Fluidigm (now Standardbiotools) 1:100
CD38 154Sm HIT2 303502 Biolegend 1:100
CD40 142Nd 5C3 3142010B Fluidigm (now Standardbiotools) 1:100
CD45 89Y HI30 3089003B Fluidigm (now Standardbiotools) 1:100
CD45RB 145Nd MEM-55 3145009B Fluidigm (now Standardbiotools) 1:100
CD49d (a4) 151Eu 9F10 304302 Biolegend 1:100
CD5 143Nd UCHT2 3143007B Fluidigm (now Standardbiotools) 1:100
CD62L (L-selectin) 153Eu DREG-56 3153004B Fluidigm (now Standardbiotools) 1:100
CD69 144Nd FN50 3144018B Fluidigm (now Standardbiotools) 1:100
CD80 (B7-1) 161Dy 2D10.4 3161023B Fluidigm (now Standardbiotools) 1:100
FcRL4 170Er 413D12 340202 Biolegend 1:100
HLA-DR 173Yb L243 3173005B Fluidigm (now Standardbiotools) 1:100
IgA 148Nd Polyclonal 3148007B Fluidigm (now Standardbiotools) 1:100
IgD 146Nd IA6-2 3146005B Fluidigm (now Standardbiotools) 1:100
IgG 171Yb G18-145 555784 BD Biosciences 1:100
IgM 172Yb MHM-88 3172004B Fluidigm (now Standardbiotools) 1:100
Integrin b7 162Dy FIB504 3162026B Fluidigm (now Standardbiotools) 1:100
RANKL 149Sm MIH24 347508 Biolegend 1:100
TACI 168Er 1A1 311902 Biolegend 1:100
CD3 160Gd UCHT1 300402 Biolegend 1:100
CD14 160Gd M5E2 3160001B Fluidigm (now Standardbiotools) 1:100

IMC PANEL 1
Marker Metal Tag Cat number Supplier Clone Dilution
1 Lysozyme 141 860001 Biolegend POLYCLONAL 200
2 CD11b 142 49420S Cell Signaling Technology D6X1N 1000
3 VIMENTIN 143 3143027D Standardbiotools D21H3 500
4 FITC-TUNEL 144 3144006B Standardbiotools FIT-22 150
5 CD38 145 ab226034 Abcam EPR4106 100
6 BTLA 146 ab254287 Abcam EPR22224-271 100
7 CD163 147 3147021D Standardbiotools EDHu-1 300
8 CD5 148 NBP2-34583 Novus Biologicals C5/473 + CD5/54/F6 200

9 IgM 149 66484-1g Fisher Scientific 2D10B10 100
10 CD11c 150 ab216655 Abcam EP1347Y 400
11 CD31 151 3151025D Standardbiotools EPR3094 300
12 CD103 152 ab254201 Abcam EPR22590-27 200
13 FCRL4 153 ab239754 Abcam EPR21961 75
14 IgD 154 ab236778 Abcam EPR6146 300
15 DNASE-I 155 ab113241 Abcam polyclonal 200
16 CD45RB 156 310202 Biolegend MEM-55 500
17 E-cad 158 3158029D Standardbiotools 24E10 3000
18 CD68 159 3159035D Standardbiotools KP1 400
19 CD20 161 3161029D Standardbiotools H1 300
20 CD8 162 3162034D Standardbiotools C8/144B 400
21 LAMP3 163 353039 Biolegend H5C6 500
22 CD1c 164 ab156708 Abcam OTI2F4 500
23 CD74 166 3166018B Standardbiotools LN2 500
24 GzB 167 3167021D Standardbiotools EPR20129-217 300
25 Ki67 168 3168022D Standardbiotools B56 400
26 PDL1 169 MAB1561 RnD Systems 130021 150
27 CD3 170 3170019D Standardbiotools POLYCLONAL 400
28 CD27 171 3171024D Standardbiotools EPR8569 300
29 CD40 172 NBP2-34488 Novus Biologicals CL1673 200
30 CD45RO 173 3173016D Standardbiotools UCHL1 500
31 NOX2 175 sc-130543 Santa Cruz 54.1 1000
32 CD83 176 ab275032 Abcam EPR23809-19 300

IMC PANEL 2
Marker Metal Tag Cat number Supplier Clone Dilution
1 Lysozyme 141 860001 Biolegend POLYCLONAL 200
2 CD11b 142 49420S Cell Signaling Technology D6X1N 1000
3 VIMENTIN 143 3143027D Standardbiotools D21H3 500
4 CD38 145 ab226034 Abcam EPR4106 100
5 BTLA 146 ab254287 Abcam EPR22224-271 100
6 CD163 147 3147021D Standardbiotools EDHu-1 300
7 IgA 148 3148007B Standardbiotools polyclonal 200
8 IgM 149 66484-1-IG-20UL Fisher Scientific 2D10B10 100
9 CD11c 150 ab216655 Abcam EP1347Y 400
10 CD31 151 3151025D Standardbiotools EPR3094 300
11 CD103 152 ab254201 Abcam EPR22590-27 200
12 FCRL4 153 ab239754 Abcam EPR21961 75
13 IgD 154 ab236778 Abcam EPR6146 300
14 DNASE-I 155 ab113241 Abcam polyclonal 200
15 CD45RB 156 310202 Biolegend MEM-55 250
16 E-cad 158 3158029D Standardbiotools 24E10 3000
17 CD68 159 3159035D Standardbiotools KP1 400
18 CD8 162 3162034D Standardbiotools C8/144B 400
19 "Digoygenin
- DNASE1L3 " 163 MAB7520 RnD Systems 611621 500
20 GzB 167 3167021D Standardbiotools EPR20129-217 300
21 Ki67 168 3168022D Standardbiotools B56 400
22 CD3 170 3170019D Standardbiotools POLYCLONAL 400
23 CD27 171 3171024D Standardbiotools EPR8569 300
24 CD40 172 NBP2-34488 Novus Biologicals CL1673 200
25 CD45RO 173 3173016D Standardbiotools UCHL1 500
26 FITC - ITGAX 174 3174006B Standardbiotools FIT-22 150
27 NOX2 175 sc-130543 Santa Cruz 54.1 750
28 CD83 176 ab275032 Abcam EPR23809-19 300

IMC PANEL 3
Marker Metal Tag Cat number Supplier Clone Dilution
1 Lysozyme 141 860001 Biolegend POLYCLONAL 200
2 CD11b 142 D6X1N Cell Signaling Technology D6X1N 1000
3 VIMENTIN 143 3143027D Standardbiotools D21H3 500
4 CD38 145 ab226034 Abcam EPR4106 100
5 BTLA 146 ab254287 Abcam EPR22224-271 100
6 CD163 147 3147021D Standardbiotools EDHu-1 300
7 IgA 148 3148007B Standardbiotools polyclonal 200
8 IgM 149 66484-1g Fisher Scientific 2D10B10 100

9 CD11c 150 ab216655 Abcam EP1347Y 400
10 CD31 151 3151025D Standardbiotools EPR3094 300
11 CD103 152 ab254201 Abcam EPR22590-27 200
12 FCRL4 153 ab239754 Abcam EPR21961 75
13 IgD 154 ab236778 Abcam EPR6146 300
14 DNASE-I 155 ab113241 Abcam polyclonal 200
15 CD45RB 156 310202 Biolegend MEM-55 250
16 E-cad 158 3158029D Standardbiotools 24E10 3000
17 CD68 159 3159035D Standardbiotools KP1 400
18 CD8 162 3162034D Standardbiotools C8/144B 400
19 "Digoygenin
- DNASE1L3" 163 MAB7520 RnD Systems 611621 500
20 "Biotin
- Bacterial 16S" 166 409002 Biolegend 1D4-C5 150
21 GzB 167 3167021D Standardbiotools EPR20129-217 300
22 Ki67 168 3168022D Standardbiotools B56 400
23 CD3 170 3170019D Standardbiotools POLYCLONAL 400
24 CD27 171 3171024D Standardbiotools EPR8569 300
25 CD40 172 NBP2-34488 Novus Biologicals CL1673 200
26 CD45RO 173 3173016D Standardbiotools UCHL1 500
27 FITC - ITGAX 174 3174006B Standardbiotools FIT-22 150
28 NOX2 175 sc-130543 Santa Cruz 54.1 750
29 CD83 176 ab275032 Abcam EPR23809-19 300

ANTIBODIES USED FOR MICROSCOPY:

-CD20- 382802 - Biolegend - 1in300
-Vimentin - 3143027D - Standard BioTools - 1in500
-CD11c- ab216655 - Abcam - 1in300
-Lysozyme - 860001 - Biolegend - 1in400

-anti-Rabbit-Alexafluor555 - A32794 - Invitrogen - 1in500
-anti-Rabitt-Alexafluor647 - A32795TR - Invitrogen - 1in500
--anti-Moure-Alexafluor647 - A32787 - Invitrogen - 1in500

Validation

Cytof antibodies were validated by the suppliers for use in staining cells in suspension experiments against human antigens. Antibodies used in the IMC panels and in the confocal microscopy experiments were validated by the suppliers as suitable for immuno-histochemistry applications in FFPE blocks. Details of the suppliers and catalogue numbers are provided above and in the Supplementary Tables. All antibodies were validated for use against human antigens. Anti-Lysozyme antibody was validated to cross-react between human and mouse (https://www.biolegend.com/en-gb/clone-search/purified-anti-lysozyme-antibody-17714?GroupID=GROUP32).

Animals and other research organisms

Policy information about studies involving animals; ARRIVE guidelines recommended for reporting animal research, and Sex and Gender in Research

Laboratory animals	No regulated procedures (including use of genetically altered animals) were carried out on the animals used for this study and so project-specific ethical approval was not required from the local Animal Welfare and Ethical Review Body (AWERB). All aspects of the housing, maintenance and culling of the mice were in accordance with the Animals (Scientific Procedures) Act 1986 and Amendment Regulations 2012.
Wild animals	N/A
Reporting on sex	N/A
Field-collected samples	N/A
Ethics oversight	N/A

Note that full information on the approval of the study protocol must also be provided in the manuscript.

Plants

Seed stocks	N/A
Novel plant genotypes	N/A
Authentication	N/A
